# Characterization of norbelladine synthase and noroxomaritidine/norcraugsodine reductase reveals a novel catalytic route for the biosynthesis of Amaryllidaceae alkaloids including the Alzheimer’s drug galanthamine

**DOI:** 10.3389/fpls.2023.1231809

**Published:** 2023-08-30

**Authors:** Bharat Bhusan Majhi, Sarah-Eve Gélinas, Natacha Mérindol, Simon Ricard, Isabel Desgagné-Penix

**Affiliations:** ^1^ Department of Chemistry, Biochemistry and Physics, Université du Québec à Trois-Rivières, Trois-Rivières, Québec, QC, Canada; ^2^ Plant Biology Research Group, Université du Québec à Trois-Rivières, Trois-Rivières, Québec, QC, Canada

**Keywords:** Amaryllidaceae alkaloids, Leucojum aestivum, Narcissus papyraceus, norbelladine synthase, noroxomaritidine/norcraugsodine reductase, enzyme activity

## Abstract

Amaryllidaceae alkaloids (AAs) are a large group of plant specialized metabolites with diverse pharmacological properties. Norbelladine is the entry compound in AAs biosynthesis and is produced from the condensation of tyramine and 3,4-dihydroxybenzaldehyde (3,4-DHBA). There are two reported enzymes capable of catalyzing this reaction *in-vitro*, both with low yield. The first one, norbelladine synthase (NBS), was shown to condense tyramine and 3,4-DHBA, while noroxomaritidine/norcraugsodine reductase (NR), catalyzes a reduction reaction to produce norbelladine. To clarify the mechanisms involved in this controversial step, both *NBS* and *NR* homologs were identified from the transcriptome of *Narcissus papyraceus* and *Leucojum aestivum*, cloned and expressed in *Escherichia coli.* Enzymatic assays performed with tyramine and 3,4-DHBA with each enzyme separately or combined, suggested that NBS and NR function together for the condensation of tyramine and 3,4-DHBA into norcraugsodine and further reduction into norbelladine. Using molecular homology modeling and docking studies, we predicted models for the binding of tyramine and 3,4-DHBA to NBS, and of the intermediate norcraugsodine to NR. Moreover, we show that NBS and NR physically interact in yeast and *in-planta*, that both localize to the cytoplasm and nucleus and are expressed at high levels in bulbs, confirming their colocalization and co-expression thus their ability to work together in the same catalytic route. Finally, their co-expression in yeast led to the production of norbelladine. In all, our study establishes that both NBS and NR participate in the biosynthesis of norbelladine by catalyzing the first key steps associated in the biosynthesis of the Alzheimer’s drug galanthamine.

## 1 Introduction

Amaryllidaceae alkaloids (AAs) are a large group of plant specialized metabolites with large therapeutical potential. The greatest commercial success among AAs is galanthamine, produced by many *Narcissus, Galanthus* and *Leucojum* species, and presently used as an acetylcholinesterase inhibitor to fight Alzheimer’s disease symptoms (Heinrich and Lee Teoh, 2004). Other AAs with strong antiviral activity, such as lycorine and cherylline, are intensively studied to fight emerging infectious diseases (Wang et al., 2014; Zhang et al., 2020; Ka et al., 2021). AAs have complex carbon skeletons and are challenging to chemically synthesize. Hence, they are often extracted directly from plants, limiting their broad usage due to the often low and variable quantity produced *in vivo*. Their massive extraction could lead to a loss in the biodiversity of the endogenous flora of some countries. One interesting alternative would be to heterologously biosynthesize them in host microorganisms, developing a sustainable platform of production, but this requires prior knowledge of the metabolic pathway. Unfortunately, much more is known about the pharmacology of AAs than about their biosynthesis. Few genes encoding biosynthetic enzymes are known and scarcely any of the accepted enzymatic reactions have been thoroughly characterized (Desgagné-Penix, 2021).

**Figure 1:**
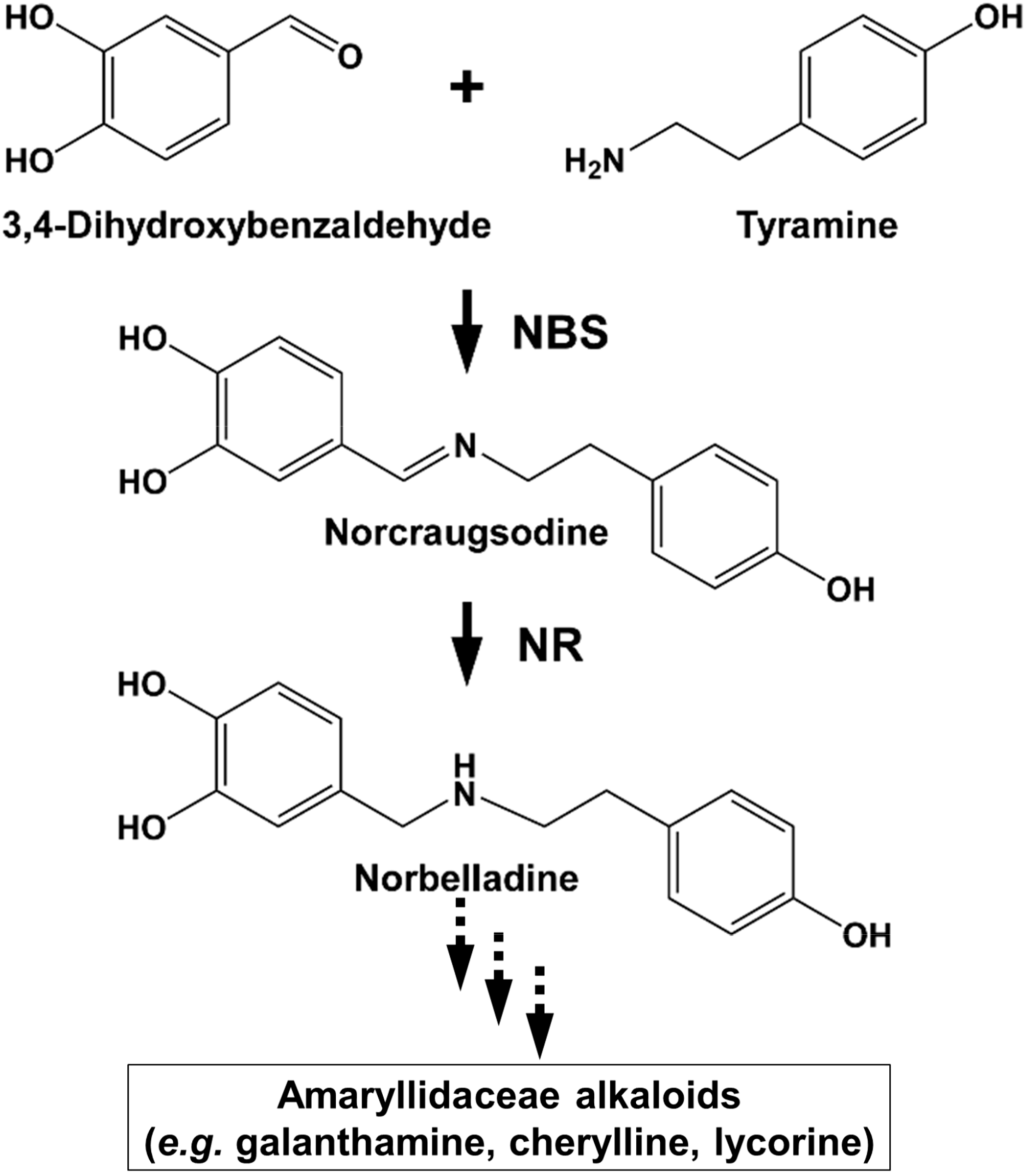
Norbelladine synthase (NBS) and noroxomaritidine/norcraugsodine reductase (NR) are involved in the proposed biosynthetic pathway for galanthamine, lycorine and haemanthamine. NBS catalyzes the condensation of tyramine and 3,4-dihydroxybenzaldehyde (3,4-DHBA) to form norcraugsodine while noroxomaritidine/norcraugsodine reductase (NR) reduces the latter to form norbelladine, the common precursor to all Amaryllidaceae alkaloids produced in plants including galanthamine, cherylline, and lycorine.

Despite their diverse chemical structures, AAs share norbelladine as a common biosynthetic origin (**Figure 1**). Following its synthesis, norbelladine undergoes several chemical modifications performed by a myriad of enzymes catalyzing various reactions, such as *O-* and *N-*methylations (OMTs, NMTs), C−C and C−O bond formation, oxidations and reductions, demethylations, and hydroxylations resulting in a array of different structural types of AAs (Eichhorn et al., 1998; El Tahchy et al., 2010; El Tahchy et al., 2011; Kilgore et al., 2014; Saliba et al., 2015; Kilgore et al., 2016a).

Previous studies have tried to unravel norbelladine biosynthesis because of its pivotal role in AAs biosynthesis. We and others identified several AA biosynthetic genes encoding enzymes involved in these early steps (Kilgore et al., 2014; Kilgore et al., 2016b; Singh and Desgagné-Penix, 2017; Singh et al., 2018; Hotchandani et al., 2019; Tousignant et al., 2022). Norbelladine originates from the condensation of tyramine and 3,4-dihydroxybenzaldehyde (3,4-DHBA), respectively derived from L-tyrosine and L-phenylalanine. The enzymes responsible for tyramine and 3,4-DHBA biosynthesis are well known and often found in most plant species (*e.g.*, tyrosine decarboxylase (TYDC), phenylalanine ammonia-lyase (PAL), cinnamate 4-hydroxylase (C4H), *etc.*). Nevertheless, it is uncertain which enzymes and intermediates specific to Amaryllidaceae are responsible for norbelladine synthesis. The currently accepted model states that the condensation of tyramine and 3,4-DHBA leads to the formation of the imine norcraugsodine, which is further reduced into norbelladine (Battersby et al., 1961). To date, there are two reported enzymes able to catalyze these reactions *in vitro*, but both perform poorly (Kilgore et al., 2016b; Singh et al., 2018). The first one is norbelladine synthase (NBS), recently characterized from two plant species *N. pseudonarcissus* (*NpKA*NBS) and *L. aestivum* (*La*NBS). NBS was shown to condense tyramine and 3,4-DHBA to form low levels of norbelladine (Singh et al., 2018; Tousignant et al., 2022). The second one, noroxomaritidine/norcraugsodine reductase (NR) from *N. pseudonarcissus* (*NpKA*NR) mainly catalyzes noroxomaritidine reduction, but also produces above background levels of norbelladine following incubation with tyramine, 3,4-DHBA and NADPH (Kilgore et al., 2016b). Therefore, the contribution of these two enzymes to norbelladine synthesis *in vivo* is still not clear.

We hypothesized that norbelladine is formed through two separate reactions (*i.e.*, condensation and reduction) catalyzed by two distinctive enzymes (**Figure 1**). In this study, we characterized the NBS and NR enzymes from two Amaryllidaceae plants species *N. papyraceus* and *L. aestivum* to try and elucidate the crucial reactions involved in norbelladine synthesis. We propose that for NBS or NR to induce norbelladine synthesis efficiently, the two-step mechanism requires the catalytic activity of both enzymes interacting together in a metabolon.

## 2 Materials and methods

### 2.1 Plant materials and growth condition

Paperwhite narcissus (*Narcissus papyraceus*) and summer snowflake (*Leucojum aestivum*) bulbs were purchased from Vesey’s (York, PE, Canada). Bulbs were planted in plastic pots using autoclaved AGRO MIX G6 potting soil (Fafard, Saint-Bonaventure, QC, Canada). The plants were kept at room temperature with exposure to tube lighting in long day (16 h of light/8 h of dark) conditions until flowering. The plants were watered when necessary to keep the soil moist. Different tissues such as bulbs, roots, stems, leaves, and flowers were collected, flash frozen in liquid nitrogen, and stored at −80°C until further used. *Nicotiana benthamiana* (Goodin et al., 2008) plants were grown in a growth chamber in long day (16 h of light/8 h of dark) conditions at 22°C.

### 2.2 Bacterial and yeast strains and growth conditions

The bacteria used in this study were *Escherichia coli* DH5α (Invitrogen), *E. coli* Rosetta (DE3) pLysS (Novagen), and *Agrobacterium tumefaciens* GV3101 (Holsters et al., 1980). The yeast strain (*Saccharomyces cerevisiae*) used is Y2HGold (Clontech Laboratories). The bacteria were grown in Luria-Bertani (LB) medium supplemented with the appropriate antibiotics at the following temperatures: *E. coli* at 37°C; and *A. tumefaciens* at 28°C. Antibiotics were used at the following concentrations (µg/mL): ampicillin, 100; chloramphenicol, 34; kanamycin, 50; rifampicin, 50; gentamicin, 30. Yeasts were grown at 30°C in selective synthetic complete medium.

### 2.3 PCR amplification, cloning, and construction of vectors

RNA was extracted from *N. papyraceus* (*Np*) and *L. aestivum* (*La*) bulbs using CTAB (cetrimonium bromide) method as described by Singh and Desgagné-Penix (2017). cDNA was synthesized from 1 µg RNA samples using SensiFAST cDNA synthesis kit (Bioline) according to manufacturer’s protocol. The open reading frame (ORF) of full length *NpNBS*, *NpNR*, *NpTR*, *LaNBS*, *LaNR*, and *LaTR* were amplified from bulbs cDNA using PrimeStar GXL premix (TaKaRa Bio) in 50 μL reaction with 0.2 μM forward and reverse primers (**Supplementary Table S2**). PCR program parameters: 2 min 98°C 1 cycle, 10 s 98°C, 20 s 55°C, 1 min 72°C for 35 cycles, 5 min 72°C 1 cycle, and final infinite hold at 4°C. A classical restriction digestion based cloning approach was used to clone and create all the desired vectors.

For protein expression in *E. coli*, *NpNBS*, *NpNR*, *NpTR (tropinone reductase)*, *LaNBS*, *LaNR*, and *LaTR* were fused to the C-terminus of the maltose binding protein (MBP) in the pMAL-c2x vector (New England Biolabs). Precisely, the full-length ORFs were amplified from cDNA by PCR using primers reported (respective restriction enzyme sites are underlined, **Supplementary Table S2**). PCR products were cleaned using GenepHlow Gel/PCR kit (Geneaid). Purified PCR products of *NpNBS*, *NpNR*, *LaNBS*, and *LaNR* were digested with *Bam*HI and *Hin*dIII, while *NpTR* and *LaTR* were digested with *Bam*HI and *Sal*I and ligated into pMAL-c2x vector digested with *Bam*HI/*Hin*dIII and *Bam*HI/*Sal*I respectively, using T_4_ DNA ligase (New England Biolabs). The recombinant plasmids were transformed into chemically competent *E. coli* DH5α cells by heat shock transformation and colonies were selected on ampicillin LB agar plates. The positive clones were identified by colony PCR in a 20 μL reaction using Taq DNA polymerase with ThermoPol buffer (New England Biolabs) with PCR parameters: 5 min 95°C 1 cycle, 30 s 95°C, 40 s 55°C, 1 min 68°C for 30 cycles, 5 min 68°C 1 cycle, and final infinite hold at 4°C. The resulting plasmids were verified by DNA sequencing to ensure the correct sequence and exclude undesired mutations.

For split luciferase complementation assays (SLCA) in *N. benthamiana* leaves, the *NpNBS*, *NpNR*, *NpTR*, *LaNBS*, *LaNR*, and *LaTR* genes were cloned into pCAMBIA1300:CLuc fused to the C-terminal (398-550 amino acids) of firefly luciferase (Cluc), and *NpNBS*, *NpNR*, *LaNBS*, and *LaNR* into pCAMBIA1300:Nluc fused to the N-terminal (2-416 amino acids) of firefly luciferase (Nluc) and driven by the CaMV 35S promoter (Chen et al., 2008). The full-length ORFs were amplified by PCR using reported primers from cDNA (respective restriction enzyme sites are underlined, **Supplemental Table S2**). After cleaning up using GenepHlow Gel/PCR kit (Geneaid), PCR products were digested with the mentioned restriction enzyme pair and ligated into the corresponding sites of pCAMBIA1300:Cluc or pCAMBIA1300:Nluc vectors using T_4_ DNA ligase. The recombinant plasmids were transformed into chemically competent *E. coli* DH5α cells and colonies were selected on kanamycin LB agar plates. The positive clones were identified by colony PCR. The resulting binary vectors were verified by DNA sequencing.

For yeast two-hybrid assays, genes encoding full-length *NpNBS*, *NpNR*, *NpTR*, *LaNBS*, *LaNR*, and *LaTR* were amplified from *N. papyraceus* and *L. aestivum* bulbs cDNA and cloned into the pGBKT7 (bait) or pGADT7 (prey) vectors (Clontech Laboratories) in frame with the GAL4 DNA binding domain (DNA-BD) or GAL4 activation domain (AD). The full-length genes were amplified by PCR using primers reported (respective restriction enzyme sites are underlined, **Supplemental Table S2**). After cleaning up using GenepHlow Gel/PCR kit (Geneaid), PCR products were digested with the mentioned restriction enzyme pair and ligated into the corresponding sites of pGBKT7 (bait) or pGADT7 (prey) vectors using T_4_ DNA ligase. The recombinant plasmids were transformed into chemically competent *E. coli* DH5α cells. Colonies with bait plasmids were selected on kanamycin while colonies with prey plasmids were selected with ampicillin LB agar plates. The positive clones were identified by colony PCR. The resulting plasmids were verified by DNA sequencing.

For subcellular localization, *NpNBS*, *NpNR*, *LaNBS*, and *LaNR* coding sequences were fused upstream to the gene encoding the yellow fluorescence protein (YFP) in the pBTEX binary vector under the control of the CaMV 35S promoter (Frederick et al., 1998). The full-length ORFs were amplified (respective restriction enzyme sites are underlined, **Supplemental Table S2**). The PCR products were cleaned up using GenepHlow Gel/PCR kit (Geneaid), digested with *Kpn*I/*Xba*I, and ligated into pBTEX-YFP vector digested with *Kpn*I/*Xba*I using T_4_ DNA ligase. The recombinant plasmids were transformed into chemically competent *E. coli* DH5α cells and colonies were selected on kanamycin LB agar plates. The positive clones were identified by colony PCR. The resulting plasmids/binary vectors were verified by DNA sequencing.

### 2.4 Expression and purification of MBP fusion proteins in *E. coli*

*NpNBS*, *NpNR*, *NpTR*, *LaNBS*, *LaNR*, and *LaTR* were cloned into the pMAL-c2x vector. The purified plasmids were transformed using heat shock transformation into chemically competent *E. coli* Rosetta (DE3) pLysS strain for protein expression. Transformed cells were selected on LB plates with ampicillin, and chloramphenicol overnight at 37°C. The positive colonies were screened by colony PCR. A PCR positive single colony was picked and grown overnight at 37°C at 220 rpm in 12.5 mL LB broth containing ampicillin and chloramphenicol. The overnight grown pre-culture was added into fresh 250 mL LB broth containing ampicillin and chloramphenicol and grown at 220 rpm at 37°C to an OD_600_ = 0.5 to 0.6. The cultures were brought to room temperature and Isopropyl-β-D-thiogalactopyranoside (IPTG) was added to a final concentration of 0.25 mM to induce protein expression. The cultures were further incubated for 20 h at 18°C at 150 rpm. Bacterial cultures were pelleted at 10,000 rpm for 15 min at 4°C, resuspended in 25 mL column binding buffer (25 mM Tris-HCl [pH 7.5], 150 mM NaCl, and 1 mM EDTA) and frozen at −80°C overnight. The cultures were thawed on ice water and 1 mM phenylmethylsulfonyl fluoride [PMSF] with 0.1x protease inhibitor cocktail (Cell Signaling Technology) was added. Cultures were then lysed using a sonicator at 41% amplitude for a total of 8 min with 15 s run and 35 s cooling time in ice. The lysates were centrifuged twice at 14,000 rpm for 20 min at 4°C to pellet the cell debris and the clear supernatants were collected. Supernatants were mixed with 500 μL of amylose resin beads (New England Biolabs) (prewashed with column binding buffer and resuspended to 50% slurry) and incubated for 1 h at 4°C with constant rocking. The mixture was passed twice through the filter columns (Thermo Scientific) with gravitational flow to retain the beads with bound proteins in the column matrix. The beads were washed with gravitational flow in the columns three times with 30 mL column binding buffer. The bead slurry was transferred to microcentrifuge tubes and centrifuged at 1000 rpm for 1 min and the supernatant was removed. Finally, the bound proteins from the bead pellet were eluted twice (elution 1 and 2) each time in 500 μL elution buffer (15 mM maltose in column binding buffer) by centrifugation at 1000 rpm for 1 min and supernatant/elute was collected. The protein samples were flash frozen in liquid nitrogen and stored at −80°C. Protein quantification was done using DC protein assay kit (Bio-Rad) according to the manufacturer’s instructions with bovine serum albumin (BSA) as standard, and protein samples were fractionated by 10% (v/v) SDS-PAGE and stained with Coomassie Blue.

### 2.5 Protein analysis and alignment

The *in-silico* protein analysis was done by DNAMAN analysis software (Lynnon BioSoft). Protein sequences of *Np*NBS, and *La*NBS were aligned with *Narcissus pseudonarcissus* ‘King Alfred’ norbelladine synthase (*NpKA*NBS; GenBank: AYV96792.1), and *Thalictrum flavum* norcoclaurine synthase (*Tf*NCS; GenBank: ACO90248.1). Similarly, *Np*NR, *La*NR, *Np*TR and *La*TR were aligned with *N. pseudonarcissus* noroxomaritidine/norcraugsodine reductase (*NpKA*NR; KU295569) using CLUSTAL W algorithm in T-Coffee software (Notredame et al., 2000) with default parameters. Sequence alignments were formatted using Boxshade program (https://embnet.vital-it.ch/software/BOX_form.html).

### 2.6 Molecular homology modelling and docking

Amino acid sequences corresponding to *Np*NBS, *La*NBS, *Np*NR, *La*NR, *Np*TR, *La*TR candidates were uploaded on Protein Homology/analogY Recognition Engine V 2.0 (Phyre2) (Kelley et al., 2015) website, I-Tasser from Zhang lab (Yang and Zhang, 2015) and MOE 2020.09 software (Chemical Computing Group) to model NBS and NR proteins. Following close analysis of predicted structures and comparison by superimposition with orthologs and homologs, the most consistent models were selected. *La*NR from I-Tasser, *Np*NR and *La* and *Np*TR models from Phyre2 were used, while NBS were best modeled by MOE. MOE was further used to analyze the resulting homology model conformation and prepare receptors for docking. First, modeled structures were compared to their template crystal structures in complex with their ligands downloaded from the Protein Data Bank (for NBS: norcoclaurine synthase from *Thalictrum flavum* in complex with dopamine and hydroxybenzaldehyde 2VQ5 (Ilari et al., 2009), NR: noroxomaritidine/norcraugsodine reductase in complex with NADP+ and tyramine 5FF9 *NpKA*NR (Kilgore et al., 2016b), TR: Tropinone reductase-II complexed with NADP+ and pseudotropine 2AE2 and 5FF9 (Yamashita et al., 1999), aligning amino-acid sequences and then superimposing the structures.

Structure preparation consisted of correcting issues, capping, charging termini, selecting appropriate alternate, and calculate optimal hydrogen position and charges using Protonate 3D. Fixed receptor and tethered active site energy minimization was performed for each modeled protein in presence of template ligands prior to docking. Ready to dock ligands were uploaded from ZINC15 (Sterling and Irwin, 2015) when available, or manually drawn (from SMILES), washed, prepared, and minimized with MOE. The MMFF94× force field was used. Receptors active site was predicted using MOE Site Finder and used as docking site to place ligand using Triangle Matcher as placement method for 200 poses and tethered induced fit as refinement to perform flexible docking. Ten resulting poses were analyzed. The most probable poses based on literature description of templates active site, on comparison with crystalized templates interactions and on scores are presented. For NBS, 3,4-DHBA was docked first, the most consistent pose was further included in the active site used for tyramine docking. Similarly for NR, NADPH was docked first, and the most consistent pose compared to template crystals was further included in the active site used to dock norcraugsodine. PLIP was used to analyze interactions between ligands and receptors (Adasme et al., 2021), and were further processed using PyMOL (Shrödinger).

### 2.7 Substrates and standards preparation

Norbelladine and norcraugsodine were synthesized as previously described (Singh et al., 2018). Standard solutions of 3,4-DHBA (Fisher Scientific), tyramine (Sigma-Aldrich), and papaverine (Sigma-Aldrich) were prepared at 1000 mg/L in LC-MS grade methanol (Sigma-Aldrich). Standard solutions of norbelladine and norcraugsodine were prepared as previously described (Singh et al., 2018). From these standard solutions, dilutions were performed to obtain working solutions of 100 mg/L in methanol, and 1 mg/L in the mobile phase (ammonium acetate 10 mM (Sigma-Aldrich) (pH 5.0), and acetonitrile (Sigma-Aldrich) [60:40]). Standards and solutions were stored in the dark at −20°C.

### 2.8 Enzymatic assays

Single enzyme assays were performed at 35°C for 2 h. All the enzymatic reactions were terminated by the addition of 10 μL of 20% trichloroacetic acid (TCA). Negative controls were purified MBP-tag protein from *E. coli*, and reactions without substrate or cofactor. The catalytic activity of purified NBS enzymes were analyzed following the method of Singh et al., 2018 with minor modifications. Reactions were conducted using 80 μg of purified proteins in 100 mM HEPES buffer (pH 6.0), with 10 μM tyramine and 300 μM 3,4-DHBA, in a total volume of 100 μL. Reaction components were equilibrated at 35°C and the reaction was started by the addition of enzyme to the substrate and buffer mixture. NR and TR single enzyme assays were performed as previously reported by Kilgore et al., (2016b) with minor modifications. The assay mix contained 60 μg of purified proteins, 10 μM tyramine, 300 μM 3,4-DHBA, and 1 mM NADPH in 100mM sodium citrate buffer (pH 6.0), in a total volume of 100 μL. The assays with NBS and NR or TR together in a single-step reaction contained 80 μg of purified NBS enzyme, 60 μg of purified NR/TR enzyme, 10 μM tyramine, 300 μM 3,4-DHBA, and 1 mM NADPH in 100mM HEPES buffer (pH 6.0), in a total volume of 100 μL. The assays with NBS and NR/TR in two-step reactions were conducted as follows: 80 μg of purified NBS enzyme in 100 mM HEPES buffer (pH 6.0), with 10 μM tyramine and 300 μM 3,4-DHBA, in a total volume of 100 μL was incubated at 35°C for 2 h. The NBS enzyme was inactivated by boiling at 95°C for 10 min, sample was centrifuged, and the supernatant (100 μL) was used as norcraugsodine solution, and 60 μg of purified NR/TR enzyme and 1 mM NADPH were added and incubated for additional 2 h. Following the reaction termination, papaverine (1000 mg/L) was added to all the reaction serving as an internal standard for the relative quantification of detected compounds. All reactions were performed in triplicates. The reaction samples were diluted 100 fold with mobile phase (ammonium acetate 10 mM [pH 5.0], and acetonitrile [60:40]) and analysis of the enzymatic product (norbelladine) using a high-performance liquid chromatography (HPLC) system coupled with a tandem mass spectrometer (MS/MS) was carried out as described by (Tousignant et al., 2022).

### 2.9 Derivatization of norcraugsodine and GC-MS analysis

Norcraugsodine (257 m/z) could not be detected by GC-MS without derivatization. For the derivatization, dry enzymatic assay samples were reconstituted in 300 μL HPLC grade acetonitrile and were transferred quantitatively in crimp-seal autosampler vials, without capping the vials. A 150 μL aliquot of BSTFA (with 1% TMCS) was added to each sample along with a magnetic stir bar, then the vials were capped using crimpers. The samples were stirred at room temperature for 60 minutes. For the GC-MS analysis, the derivatized samples were injected into the GC-MS (Agilent Technologies 6890N GC coupled with 5973N inert MSD) in electron ionization mode at 70 eV. The temperature ramp used is described as follows: temperature was set at 100°C for 2 min, followed by 100–180°C at 15°C min^−1^, 180–300°C at 5°C min^−1^, and a 10 min hold at 300°C. Injector and detector temperatures were set at 250°C and 280°C, respectively, and the flow rate of carrier gas (He) was 1 mL min^−1^. A split ratio of 1:10 was applied, and the injection volume was 1 μL. The presence of norcraugsodine in the tested samples was determined by comparison with the GC-MS analysis of a norcraugsodine standard derivatized following the same protocol. Tris-derivatized norcraugsodine (473 m/z) was observed at a retention time of 24.79 minutes and showed characteristic fragment ions (458 m/z and 294 m/z) that we respectively attributed to the loss of a methyl radical and to the loss of a 4-((trimethylsilyl)oxy)benzyl radical. The corresponding bis-derivatized aldehyde (282 m/z) was observed at a retention time of 9.28 minutes and showed characteristic fragment ions (267 m/z and 193 m/z) that we respectively attributed to the loss of a methyl radical and to the loss of a (trimethylsilyl)oxy radical.

### 2.10 *Agrobacterium*-mediated transient expression

The YFP- and LUC-fusion binary vectors were transformed into *A. tumefaciens* strain GV3101 by electroporation and colonies were selected on LB agar plates with rifampicin, kanamycin, and gentamicin at 28°C. The positive colonies were confirmed by colony PCR using PCR parameters: 10 min 95°C 1 cycle, 30 s 95°C, 40 s 55°C, 1 min 68°C for 30 cycles, 5 min 68°C 1 cycle, and a final infinite hold at 4°C. For transient expression, cultures of *A. tumefaciens* were grown overnight in 5 mL LB broth with rifampicin, kanamycin, and gentamicin at 28°C. The cultures were pelleted at 8000 rpm for 5 minutes at room temperature, washed three times with 10 mM MgCl_2_, resuspended in 5 mL of induction medium (10 mM MgCl_2_, 10 mM MES [pH 5.6], and 200 µM acetosyringone), and incubated at 28°C with shaking at 200 rpm for 3−4 h. *A. tumefaciens* cultures were diluted in the induction media to OD_600_ = 0.25 and infiltrated into young but fully expanded leaves of five-week-*old N. benthamiana* plants using a 1 mL needleless syringe. After agroinfiltration the plants were incubated in a growth chamber in long day (16 h of light/8 h of dark) conditions at 22°C for 48 h until leaf discs were harvested.

### 2.11 Split luciferase complementation assay

*NpNBS*, *NpNR*, *NpTR*, *LaNBS*, *LaNR*, and *LaTR* genes were cloned in frame to firefly luciferase fragments in the binary vector pCAMBIA1300:NLuc or pCAMBIA1300:CLuc. The obtained binary vectors were transformed into *A. tumefaciens*. The desired NLuc- and CLuc-fusion *A. tumefaciens* combinations were mixed at 1:1 ratio (OD_600_ = 0.25) and co-expressed in *N. benthamiana* leaves. Split luciferase complementation assays were performed as described by (Chen et al., 2008) with minor modifications. Three millimeter-diameter leaf discs were harvested 48 h after agroinfiltration and floated abaxial side up in 100 µL of degassed water on a white 96-well plate. Samples were supplemented with 1 mM D-luciferin (Sigma-Aldrich) and incubated in the dark for 2 min with gentle shaking and an additional 8 min at rest to quench fluorescence. Luminescence was measured using a Synergy H1 Microplate reader (BioTek) with integration time set to 2 s and imaged using a Gel Doc XR system (Bio-Rad).

### 2.12 Yeast two-hybrid analysis

*NpNBS*, *NpNR*, *NpTR*, *LaNBS*, *LaNR*, and *LaTR* genes were either fused to the GAL4 DNA binding domain (DNA-BD) in the bait vector pGBKT7 or were fused to the GAL4 activation domain (AD) in the prey vector pGADT7 (Clontech Laboratories). The yeast strain Y2H Gold (Clontech Laboratories) was first transformed with the bait vectors (*i.e.*, *NBS*, *NR*, and *TR* in the pGBKT7 vector) and subsequently with prey vectors (*i.e.*, *NBS*, *NR*, and *TR* in the pGADT7 vector). The transformants were selected on synthetically defined (SD) medium lacking leucine and tryptophan (SD-LW). The interactions were verified by testing the activation of the *HIS3*, *ADE2* and *AUR1-C* reporter genes on selective media plates lacking histidine and adenine (SD-LWHA) or containing the antibiotic Aureobasidin A (AbA), respectively.

### 2.13 Expression of NBS and NR in yeast

*NpNBS* was amplified and digested with *Bam*HI and *Hin*dIII and ligated into a similarly digested pESC-LEU vector (Stratagene) to produce pESC-LEU : *NpNBS*. Similarly, *NpNR* and *NpTR* were amplified and digested with *Bam*HI/*Hin*dIII and *Bam*HI/*Sal*I and ligated into a similarly digested pESC-LEU vector to produce pESC-LEU : *NpNR* and pESC-LEU : *NpTR*, respectively. To produce pESC-LEU : *NpNBS*-*NpNR*, the *NpNR* ORF was ligated into pESC-LEU : *NpNBS* plasmid vector digested with *Spe*I/*Bgl*II, with NBS expressed under control of the Gal1 promoter and NR under the Gal10 promoter. Yeast (INVSc1; Invitrogen) was transformed with all the above constructs and selected on SD-LEU plate for 3 d at 28°C. A single colony was used to inoculate 3 mL of SD-LEU glucose medium and incubated with shaking at 28°C for 2 d. A 500-μL aliquot of starter culture was then used to inoculate to 10 mL of SD-LEU galactose medium containing 250 μM tyramine and 250 μM 3,4-DHBA and incubated with shaking at 28°C for 3 d. A culture lacking substrates was used as a control. The cultures were centrifuged, the medium and cell pellet were extracted with 5 mL ethyl acetate, and the extracts were dissolved in mobile phase (100 μL) of (ammonium acetate 10 mM [pH 5.0], and acetonitrile [60:40]). Papaverine (1000 mg/L) was added to all the reaction as an internal standard for the relative quantification. The analysis of the product (norbelladine) was done using a high-performance liquid chromatography (HPLC) system coupled to a tandem mass spectrometer (MS/MS) as described by (Tousignant et al., 2022).

### 2.14 Protein extraction

For protein extraction from *N. benthamiana* leaves, five leaf discs (1 cm diameter) were frozen in liquid nitrogen, homogenized in 300 µL extraction buffer (100 mM Tris [pH 7.5], 1% [v/v] Triton X-100, 1 mM PMSF, and 0.1x protease inhibitor cocktail), and centrifuged at 17,000 g for 30 min at 4°C. The clear supernatant was collected, and protein concentration was determined using DC protein assay kit (Bio-Rad) according to the manufacturer’s instructions.

For protein extraction from yeast, 5 mL overnight-grown cultures were pelleted at 12,000 g for 5 min at 4°C, resuspended in 250 µL ice-cold lysis buffer (4% [v/v] 5 N NaOH and 0.5% [v/v] β-mercaptoethanol), and incubated with 1x SDS sample buffer (30% [v/v] glycerol, 15% [v/v] β-mercaptoethanol, 37.5% [v/v] 500 mM Tris-HCl [pH 6.8], 0.15% [w/v] SDS, and a few grains of Bromophenol Blue) for 10 min at 95°C.

### 2.15 Western blotting

Equal amounts of protein (100 µg) were fractionated by 10% (v/v) SDS-PAGE. Proteins from gels were transferred onto Polyvinylidene difluoride (PVDF) membrane using Trans-Blot Turbo transfer system (Bio-Rad). The membrane was equilibrated with Tris-buffered saline (TBS) buffer (20 mM Tris, 150 mM NaCl pH 7.6) for 15 min, followed by blocking of membrane for 2 h with TBS buffer containing 0.1% tween 20 (TBST), and 5% skim milk. The PVDF membrane was incubated overnight at 4°C in TBST with 5% milk containing 1:1000 dilution of specific primary antibodies. The primary antibodies used in this study are rabbit anti full-length firefly luciferase antibodies (Sigma-Aldrich), which react with both the N-terminal and C-terminal firefly LUC fragments, mouse anti-GFP/CFP/YFP monoclonal antibody (Cedarlane labs), mouse anti-Myc/c-Myc monoclonal antibody (Santa Cruz Biotechnology), and mouse anti-HA-tag monoclonal antibody (GenScript). After primary antibody incubation, the membrane was washed three times each for 5 min in TBST buffer and incubated for 30 min in TBST containing 5% skim milk and goat anti-rabbit horseradish peroxidase (GAR)-HRP or goat anti-mouse horseradish peroxidase (GAM)-HRP conjugate in 1:10,000 dilutions. The immunoblot was washed three times for 5 min each in TBST buffer and developed using clarity Western ECL substrate (Bio-Rad). Finally, the membrane was washed twice with TBST and stained with Ponceau S stain [0.5% (w/v) Ponceau S (Sigma-Aldrich) in 1% (v/v) acetic acid] for 1 min and photographed using Gel Doc XR system (Bio-Rad).

### 2.16 Subcellular localization

To visualize *Np*NBS, *Np*NR, *La*NBS, and *La*NR subcellular localization, the YFP fusion proteins were expressed via *A. tumefaciens* in leaves of 5-week-old *N. benthamiana* plants. Forty-eight hours post infiltration, the abaxial epidermis of leaf discs were placed on a microscopic slide in a water drop, covered by a cover slip, and imaged immediately. Protein localization was visualized by a confocal laser scanning microscope (Leica TCS SP8; Leica Microsystems) with a 40X/1.30 oil immersion objective. Images were first processed with Las AF Lite software (Leica Microsystems). CFP was used as a control for colocalization (Kruse et al., 2010). YFP was excited with an argon laser at 488 nm, while CFP was excited with a diode laser at 405 nm. Emission was detected with a spectral detector set between 500 and 525 nm for YFP and between 420 and 490 nm for CFP. Chlorophyll autofluorescence was observed with an excitation wavelength of 552 nm and the emission of fluorescence signals were detected from 630 to 670 nm. The combined images were generated using the Las X software (Leica Microsystems).

### 2.17 RNA extraction and Real-time quantitative PCR

Total RNA was isolated from bulbs, roots, stems, leaves, and flowers using the TRIzol reagent (Invitrogen). Briefly, 100 mg of tissues were frozen in liquid nitrogen, fully ground, and homogenized in 1 mL of TRIzol using a mortar and pestle. The liquid was transferred to a microcentrifuge tube, incubated 5 min at room temperature and extracted with 200 µL chloroform. Following centrifugation at 12,000 g for 15 min at 4°C, the upper phase containing RNA was transferred to a fresh tube. The RNA was precipitated with 500 µL of isopropanol for 10 min at room temperature and centrifuged at 12,000 g for 10 min at 4°C. The RNA pellet was washed twice with 1 mL of 75% ethanol (with DEPC water) and centrifuged at 7500g for 5 min at 4°C. Finally, the RNA pellet was air dried and suspended in 40 µL of DEPC-treated water. The quality and quantity of RNA extracted from different tissues were verified on NanoPhotometer (Implen) and 1.5% (w/v) agarose gel electrophoresis. RNA samples (1 µg) were reverse transcribed using SensiFAST cDNA synthesis kit (Bioline) according to manufacturer’s protocol and subjected to Real-time quantitative PCR (RT-qPCR) using gene-specific primers (**Supplementary Table S2**). The experiments were performed in triple technical replicates of each plant sample. A total reaction volume of 20 μL containing 1x SensiFAST SYBR Lo-ROX mix (Bioline), 200 μM of each forward and reverse primer, and 2 µL of template cDNA (50 ng/µL) was used for RT-qPCR analysis. RT-qPCR was performed on CFX Connect Real-Time PCR System (Bio-Rad). Amplification conditions were 95°C for 3 min 1 cycle, 95°C for 10 s, and 60°C for 30 s for 40 cycles followed by dissociation step 95°C for 10 s, 65°C for 5 s and 95°C for 5 s. *LaGAPDH* and *NpHISTONE* were used as internal reference genes for *La* and *Np*, respectively. To verify the specificity of the primers, a melting-curve analysis was also performed. The threshold cycle (C_T_) value of each gene was normalized against the C_T_ value of the reference genes. Mean C_T_ values calculated from the technical triplicates were used for quantification of relative gene expression involving the comparative C_T_ method (Pfaffl, 2001). The results were analyzed, and the statistical error was calculated using CFX Maestro software (Bio-Rad).

### 2.18 Accession numbers

Sequence data from this article can be found in GenBank under the following accession numbers: *N. papyraceus* norbelladine synthase (*NpNBS*; MZ054104), *N. papyraceus* noroxomaritidine/norcraugsodine reductase (*NpNR*; MF979872), *N. papyraceus* histone (*NpHistone*; MF979875), *L. aestivum* norbelladine synthase (*LaNBS*; MW971977), *L. aestivum* noroxomaritidine/norcraugsodine reductase (*LaNR*; MW971981), *L. aestivum* glyceraldehyde-3-phosphate dehydrogenase (*LaGAPDH*; MW971984), *Narcissus pseudonarcissus* ‘King Alfred’ norbelladine synthase (*NpKANBS*; AYV96792), *N. pseudonarcissus* noroxomaritidine/norcraugsodine reductase (*NpKANR*; KU295569).

## 3 Results

### 3.1 Identification and structure analysis of NBS homologs from *N. papyraceus* and *L. aestivum*

**Figure 2:**
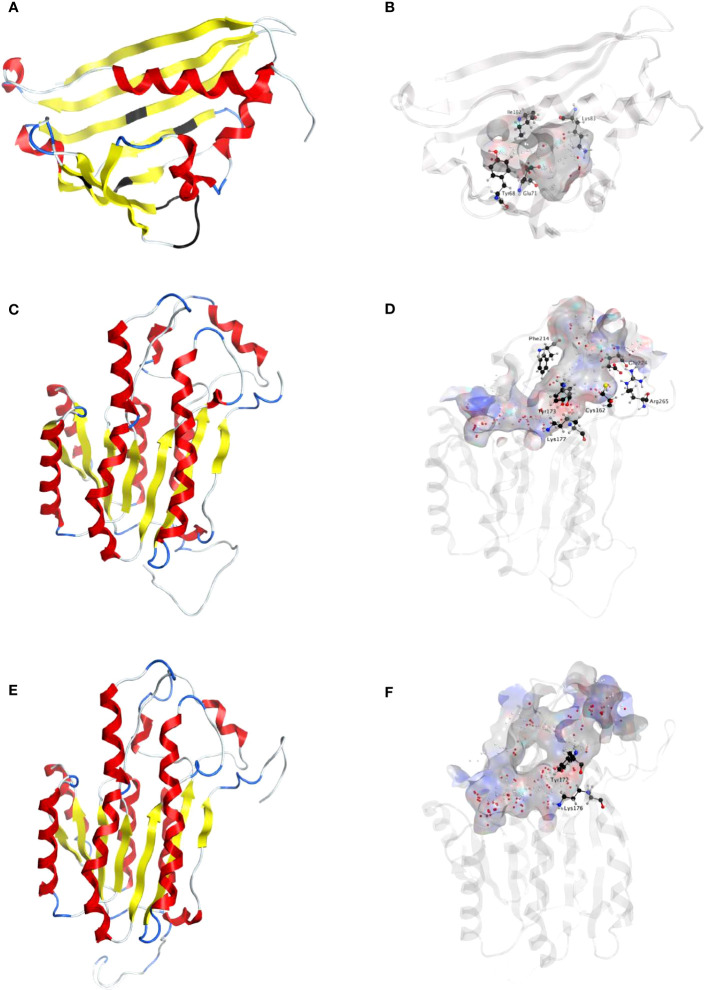
Homology modeling of *Np*NBS, NR and TR. **(A)** Ribbon representation of *Np*NBS with colored secondary structures. β-sheets are displayed yellow, α-helices red, and loops blue. Conserved glycine rich P-loop of PR10-/Betv1 enzymes is shown black, as are conserved active site residues of norcoclaurine synthase Tyr68 (Tyr108 from *Tf*NCS), Glu71 (Glu111), Lys83 (Lys123). As *Tf*NCS, NBS are composed by seven-stranded antiparallel β-sheets, two long C- and N-terminal helices and two short ones, enclosing a cleft with polar residues at its entrance and hydrophobic residues in its core. **(B)** Transparent ribbon representation of *Np*NBS with predicted active site pocket in transparent surface. The predicted ligand site computed by the Site Finder tool of MOE software is displayed as white and red alpha sphere centers inside the pocket. *Np*NBS cavities is predicted to contain an active site of 27 residues, surrounded by catalytic Tyr68, Glu71, Lys83 and Ile102 (shown as black sticks), with Tyr68 at its entrance, Lys83 at the other side and the P-loop at the bottom. **(C)** Ribbon representation of *Np*NR with highlighted secondary structures. β-sheets are displayed yellow, α-helices red and loops in blue. **(D)** Cartoon ribbon representation of *Np*NR with transparent surface view of the predicted active site forming a catalytic tunnel that crosses the enzyme. The predicted ligand site is displayed as white and red alpha sphere centers inside the pocket. *Np*NR tunnel active site is predicted to contain 55 residues. Conserved active site residues Cys162, Tyr173, Lys177, Phe214, Glu224, Arg265 surrounding the tunnel are shown as black sticks. In general, amino acids involved in NADPH binding by noroxomaritidine/norcraugsodine reductase (*NpKA*NR, 5FF9) are conserved and similarly oriented, *i.e.*, Val81 (Val83 for *NpKA*NR); Asp80 (82), Arg55 (57), Ser54 (56), Thr32 (34), catalytic residue Tyr173 (175), catalytic residue Lys177 (179), Asn108 (110); Thr208 (210), Pro203 (205), Gly204 (206) and Ala205 (207). **(E)** Ribbon representation of *Np*TR with secondary structures. β-strands are displayed yellow, α-helices red, and loops blue. **(F)** Ribbon representation of *Np*TR with transparent surface view of predicted active site tunnel crossing the enzyme. Predicted ligand site is displayed as white and red alpha sphere centers. Conserved catalytic Tyr172 and Lys176 are shown as black sticks.

The full-length cDNAs of *NpNBS*, *LaNBS*, *NpNR*, and *LaNR* candidate genes were obtained from previously reported transcriptome sequencing of *N. papyraceus* and *L. aestivum* (Hotchandani et al., 2019; Tousignant et al., 2022). The open reading frame (ORF) of both *NpNBS* and *LaNBS* gene is 480 bp and encodes a 159-amino acid protein (**Figure S1**). *In silico* protein analysis by DNAMAN analysis software indicated that *Np*NBS and *La*NBS candidates had a predicted molecular weight (MW) of 17.4 kDa and a theoretical isoelectric point (pI) of 5.3 and 5.1, respectively. Multiple amino acid sequence alignments of *Np*NBS and *La*NBS candidates with the already characterized protein *NpKA*NBS (Singh et al., 2018) and norcoclaurine synthase from *T. flavum* (*Tf*NCS) (Ilari et al., 2009) showed that *Np*NBS and *La*NBS share over 41% of amino acids sequence identity with the ortholog *Tf*NCS (**Figure S1**). In addition, *NpKA*NBS, *Np*NBS and *La*NBS share 83% identity between each other, while *Np*NBS and *La*NBS share 85% of identity (**Figure S1**). Domain search using *NCBI-conserved-domain-search service* revealed the presence of conserved Bet v1 and Pathogenesis-Related (PR-10) protein domains in both *Np*NBS and *La*NBS homologs. Both contained the phosphate-binding loop (P-loop) glycine-rich region (**Figure S1**), a conserved ligand-binding domain of Bet v1 protein family (Fernandes et al., 2013). As reported previously, the alignment showed that *Tf*NCS catalytic residues Tyr108, Glu110 and Lys122 are well-conserved in *Np*NBS and *La*NBS, corresponding to Tyr68, Glu71, and Lys83, respectively, in their sequences (**Figure S1**) (Ilari et al., 2009; Singh et al., 2018). The fourth catalytic residue Asp141 from *Tf*NCS is replaced by hydrophobic Ile102 in both *Np*NBS and *La*NBS, as reported previously for *NpKA*NBS (Singh et al., 2018). Homology modeling of the enzymes from both *N. papyraceus* (*Np*) and *L. aestivum* (*La*) revealed a striking structure similarity with superimposed template crystal of *Tf*NCS, analogous to the overall structure of Bet v1-like proteins family (**Figures 2A**; **S2A**; **S3A-D**). As *Tf*NCS, both NBS homologs are composed by seven-stranded antiparallel β-sheets, two long C- and N-terminal helices and two short ones, enclosing a cleft with polar residues at its entrance and hydrophobic residues in its core (**Figures 2A, B**; **S2A, B**).

### 3.2 Identification and structure analysis of NR homologs from *N. papyraceus* and *L. aestivum*

The ORF of *NpNR* and *LaNR* candidate genes is 810 bp, both encoding a 269-amino acid protein (**Figure S4**). *Np*NR and *La*NR homologs have a predicted molecular weight (MW) of 29 kDa and theoretical pI of 6.0 and 5.4 respectively. Sequence comparison and domain search confirmed the presence of conserved short-chain dehydrogenases/reductases (SDR) domain in *Np*NR and *La*NR candidates. *NpKA*NR, *Np*NR and *La*NR candidates share over 76% of identity with each other, while *Np*NR and *La*NR share 90% of identity (**Figure S4**). Like all classical SDRs, *Np*NR and *La*NR contain a TGXXX[AG]XG cofactor binding motif and a YXXXK active site motif, with the Tyr and Lys of the active site serving as critical catalytic residues (**Figure S4**). Structurally, predicted models of NRs from both species are formed by a seven-stranded parallel β-sheet inserted between a pair of three α-helices (**Figures 2C**; **S2C**). A long tunnel is shaped at the C-termini of β-strands partially wrapped by the α-helices and loops that elevate beyond the β-sheets (**Figures 2C, D**; **S2C, D**). At the site of ligand interaction, the tunnel expands into a larger pocket where aromatic Phe214 (216 in *NpKa*NR) is conserved, Ala112 replaces Tyr114, both possibly involved in polycyclic substrate orientation and binding. At the extremity, Glu224 (226), Arg265 (267), Cys162 (164), His170 (172) are preserved. A strong similarity in structure of both *Np*NR and *La*NR homologs with *N. pseudonarcissus* noroxomaritidine/norcraugsodine reductase (*NpKA*NR, 5FF9) was noted (**Figures S5A-D**).

To identify other reductases that could catalyze similar reduction reactions, we searched for homologs of NR in the transcriptome sequences of *N. papyraceus* (Hotchandani et al., 2019) and *L. aestivum* (Tousignant et al., 2022), and identified a candidate tropinone reductase (TR) belonging to the same SDR superfamily in both species. The ORF of the *NpTR* homolog is 825 bp and encodes a 274-amino acid protein while the *LaTR* is 816 bp and encodes a 271-amino acid protein (**Figure S4**). *Np*TR and *La*TR candidates have a predicted molecular weight (MW) of 30 kDa and 29.5 kDa and theoretical pI of 6.9 and 8.4 respectively. *Np*TR and *La*TR contain the TGXXX[AG]XG cofactor binding motif and the YXXXK catalytic active site motif (**Figure S4**). Gly206 is replaced by a tryptophan at position 203 and 200 in *Np*TR and *La*TR respectively, although this residue was conserved in active SDR/tropinone reductases (Roth et al., 2018). Multiple sequence alignments showed that *NpKA*NR, *Np*NR, *La*NR, *Np*TR, and *La*TR share over 58% of identity between each other. Predicted TRs structures are similar to NRs with some key differences in the ligand active site, including replacement of Phe216 from *NpKA*NR by Arg213 in *Np*TR and by Leu210 in *La*TR, while Tyr114 is replaced by Asn111 in *Np*TR and Asn108 in *La*TR (**Figures 2E, F**; **S2E, F**; **S6**; **Table S1**).

### 3.3 Predicted interactions of ligands with NBS and NR

**Figure 3:**
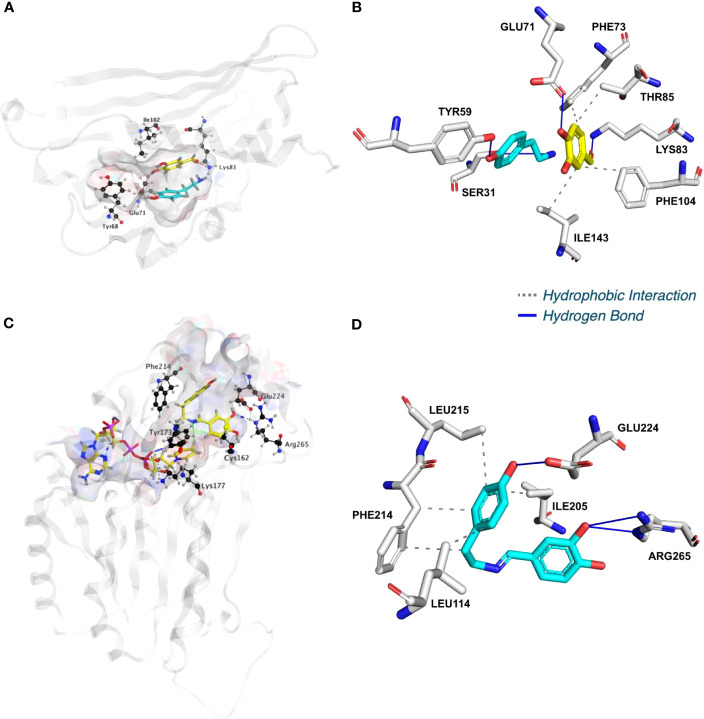
*Np*NBS docked with 3,4-dihydroxybenzaldehyde (3,4-DHBA) and tyramine. **(A)** Cartoon representation of *Np*NBS with transparent surface-active site pocket docked with 3,4-DHBA (up) and tyramine (down). Conserved catalytic residues Tyr68 (Tyr108 from *Tf*NCS), Glu71 (Glu110), Lys83 (Lys122) and Ile102 (instead of Asp141) shaping the binding site are shown as black sticks. **(B)** PLIP predicted conformation of interacting residues of *Np*NBS (grey) docked with 3,4-DHBA (turquoise) and tyramine (yellow). **(C)** *Np*NR docked with NADPH and norcraugsodine. Ligands NADPH (left) and norcraugsodine (right) are represented as thick yellow sticks. Conserved active site residues Cys162, Tyr173, Lys177, Phe214, Glu224 and Arg265) shaping the binding site are shown as thin black sticks. **(D)** PLIP predicted interacting residues of docked norcraugsodine (turquoise) with *Np*NR (grey sticks).

To shed light on the reactions involved in norbelladine synthesis (**Figure 1**), we studied the interaction of NBS and NR candidates with their respective proposed ligands through molecular docking analysis *in silico*. Tyramine and 3,4-DHBA were docked with scores of -5.03 and -5.23 kCal/mol inside the NBS pocket respectively (**Figures 3**; **S7**; **Table S1**). Most of the obtained poses displayed the same ligand orientations where 3,4-DHBA and tyramine adopted a stack configuration with their aromatic rings lying on near-to-parallel planes, similarly to dopamine and hydroxybenzaldehyde in crystalized *Tf*NCS (2VQ5) (**Figures 3A, B**; **S7A, B**). The carbonyl group of 3,4-DHBA faced the amine group of tyramine. Lys83, whose proposed role is to intercept the carbonyl group of the aldehyde substrate, interacted with 3,4-DHBA carbonyl end through hydrogen bonding (**Figure 3B**; **Table S1**). At the other end, the hydroxyl group of C4 was hydrogen-bonded with the possibly base-acting residue Glu71. All predicted poses implied interaction with Glu71 and Lys83 strengthening the probability of their key role in the catalytic mechanism. Tyramine was hold in place by stacking interaction, and hydrogen bonding between its amine and 3,4-DHBA carbonyl group. PLIP software (Adasme et al., 2021) predicted additional hydrophobic interactions between Phe73, Thr85, Phe104 and Ile143, and 3,4-DHBA, as well as two hydrogen bonds between tyramine and Ser31 and Tyr59 (**Figures 3B**; **S7B**; **Table S1**). In general, these predicted interactions and spatial arrangements of the ligands inside NBS are consistent with the reaction proposed by Ilari et al., 2009 that would lead to norcraugsodine biosynthesis.

Following its formation, norcraugsodine would be transferred from NBS to NR active site to be reduced into norbelladine (**Figure 1**) with NADPH as the electron donor. Upon docking, NADPH positioned into *La*NR and *Np*NR modeled active site following a similar arrangement compared to crystalized reductases such as 5FF9 (*NpKA*NR) with a score of -11.51 kCal/mol (**Figures 3C, D**; **S7C, D**; **Table S1**). It interacted with Gly36, Cys53, Arg55, Val109, Ile35, His170, Thr111, Lys177, Asn108, Thr206. PLIP confirmed these interacting residues and additionally predicted hydrophobic interaction with Ile35, hydrogen bonding with Lys33, Cys79, Gly110, Thr208, Val209 and Gln210 along with π-Cation interactions and salt bridges with Arg55 (**Table S1**). Close to the ligand-binding site, the nicotinamide ring faces Ile158, Pro203, Gly204 on one side, and the substrate-binding pocket on the B-side. As it was the case for docked noroxomaritidine in the active site of noroxomaritidine reductase, docking predicted that norcraugsodine binds to the active site of NR by a combination of polar and non-polar interactions. Docked norcraugsodine displayed two possible conformations in *Np*NR and *La*NR: either bended or diagonal. For both conformations, the amine group of norcraugsodine was positioned close to C4 of NADPH and to Tyr173, obtaining a docking score of -6.36 and -6.15 kCal/mol for *NpNR* and *La*NR respectively (**Figures 3C, D**; **S7C, D**; **Table S1**). In both cases, norcraugsodine phenol cycle was located near Phe214, and its dihydroxybenzene group was positioned close to Glu224, interacting with His170 and Arg265. PLIP predicted additional hydrophobic interactions of norcraugsodine with Leu114, Ile205, Phe214 and Leu215, and hydrogen-bonding with Glu224 (**Figures 3C, D**; **S7C, D**; **Table S1**). These interactions are consistent with the proposed reduction of the imine functional group of norcraugsodine to yield norbelladine by a mechanism involving NADPH and the catalytic residues Tyr173 and Lys177.

### 3.4 NBS and NR produce higher titers of norbelladine together than separately

**Figure 4:**
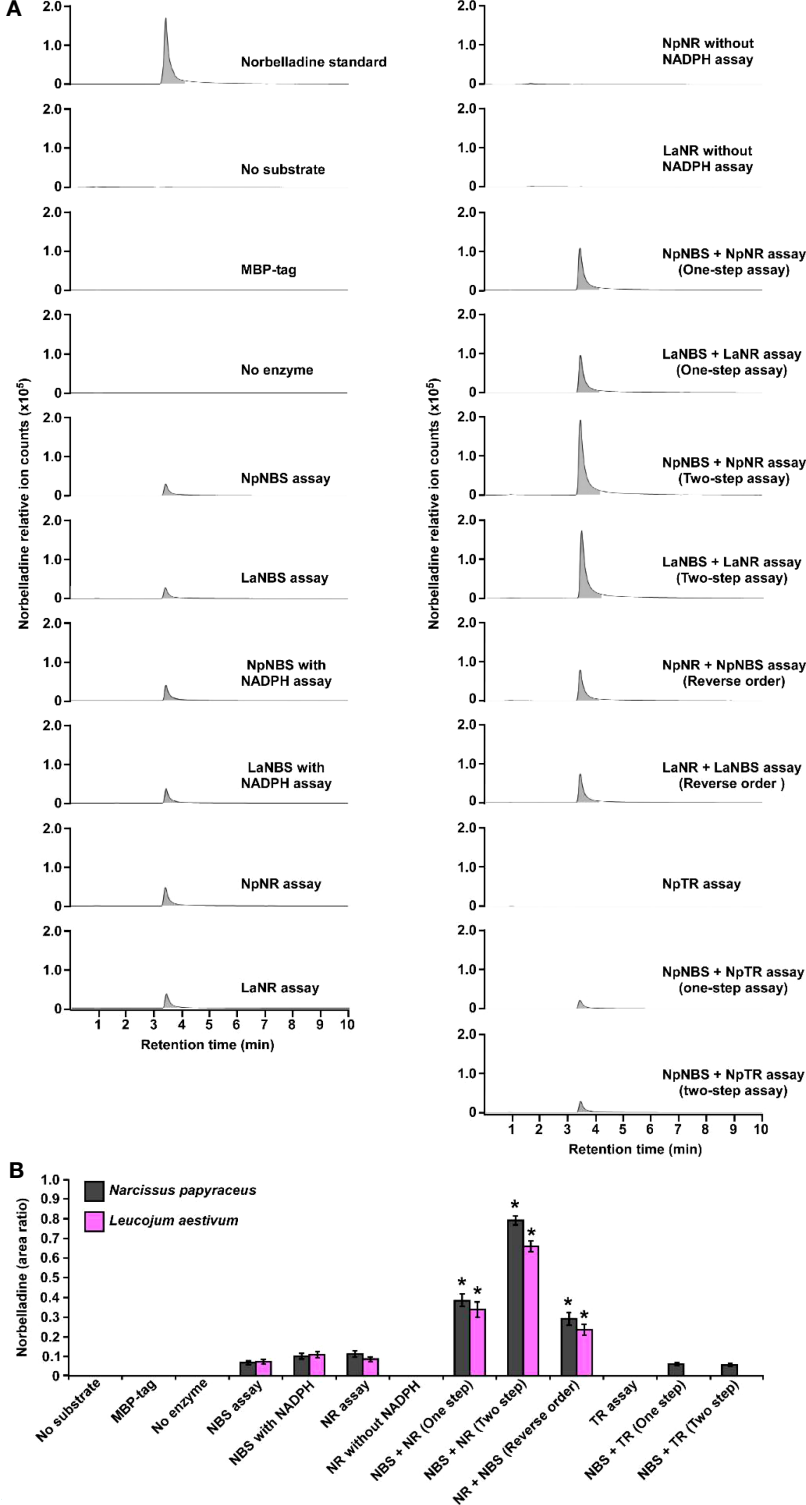
Enzymatic activity of NBS, NR and TR. Enzymes were tested separately or together for production of norbelladine, and the reaction product was monitored using HPLC-MS/MS. **(A)** Extracted ion chromatograms of quantifier MRM transition 260 → 138 m/z showing the product norbelladine in different enzymatic assays. The tested substrates used were 3,4-DHBA (300 μM) and tyramine (10 μM), and panels show norbelladine standard; assay without substrates; assay with MBP tag; assay without enzyme; and the complete assay performed with recombinant *Np*NBS, *La*NBS, *Np*NR, *La*NR and NpTR recombinant enzymes as indicated. Parent ion mass-to-charge (m/z) of 260 for norbelladine was subjected to collision-induced dissociation using multiple reaction monitoring (MRM) analysis. **(B)** Comparison and relative quantification of assays shown in **Figure 4A** in triplicate (mean ± SD, n = 3). The norbelladine product profiles in different assays performed were analyzed by HPLC-MS/MS and the obtained amount were relatively quantified using the area ratio of norbelladine produced in the assay to the papaverine internal standard. Data are means ± *SE* of three biological repeats. Asterisks indicate a significant difference (Student’s *t* test, *p* < 0.05) relative to *Np*NBS alone enzymatic assay.

To examine the NBS, NR, and TR candidate protein function from both *N. papyraceus* and *L. aestivum*, the full-length ORFs were PCR-amplified from *N. papyraceus* and *L. aestivum* bulb cDNA, cloned, and expressed proteins were purified (**Figure S8**). We were unable to purify the *La*TR enzyme in our experimental conditions. We first tested the NBS, and NR purified proteins from *N. papyraceus* (*Np*) and *L. aestivum* (*La*) separately in assays containing tyramine, 3,4-DHBA, and NADPH. The resulting assay products were subjected to HPLC-MS/MS analysis using positive electrospray ionization mode (ESI+). Before injecting the assays, norbelladine standard was injected at 1 mg/L and predicted parent-ion was observed with a mass-to-charge ratio (m/z) of 260 [M + H]+ at 3.435 min (**Figures 4A**; **S9A-R**). Fragmentation of the norbelladine parent-ion yielded to major ion fragments of m/z 121, 123 and 138 using 10 V as collision energy. Multiple reaction monitoring (MRM) transitions of 260 → 138 m/z and 260 → 121 m/z were selected, optimized using MassHunter Optimizer software, and used as quantifier and qualifier ions respectively. We could not observe any parent-ion mass for the norcraugsodine standard, despite repeated trials. We inferred that norcraugsodine was highly unstable in solution and/or thermolabile so the heat used during the ionization process in the HPLC-MS/MS source could lead to its degradation. Enzyme assays containing recombinant NBS candidate protein from *Np* or *La*, 3,4-DHBA, tyramine, and with or without NADPH yielded a peak at 3.435 min in MRM acquisition mode on HPLC-MS/MS which was the same retention time as norbelladine standard (**Figures 4A**; **S9E, F**). Similarly, for the assays examining the NR candidates, tyramine and 3,4-DHBA were incubated with NR and NADPH, and the resulting product showed a peak with the same retention time as norbelladine standard in the reaction mixture of *Np* enzyme or *La* enzyme (**Figures 4A**; **S9G, P**). Comparison of the mass spectrum obtained after the fragmentation of authentic norbelladine standard using collision-induced-dissociation (CID) with 10V and the mass spectrum of the products obtained from NBS and NR reactions showed fragmentation patterns to be the same for both, thus confirming the identity of the enzymatic product (**Figure S10**). In assays lacking substrates or enzyme, no norbelladine was detected (**Figures 4A**; **S9B, D**). Similarly, MBP tag alone protein purified from *E. coli* transformed with empty pMAL-c2X vector showed no activity (**Figures 4A**; **S9C**). The papaverine internal standard (1000 mg/L) was used to normalize each signal obtained by LC-MS/MS to have accurate relative quantification for the produced norbelladine. The relative quantification was made by comparing peak area ratios (i.e., the peak area of norbelladine divided by the peak area of papaverine internal standard) of different samples with each other. As expected, NR assays lacking NADPH resulted in no norbelladine production (**Figures 4A**; **S9C**). As reported before (Kilgore et al., 2016b; Singh et al., 2018; Tousignant et al., 2022), our results confirm that both NBS and NR homologs alone from *N. papyraceus* and *L. aestivum* are able to catalyze the reaction of condensation/reduction of tyramine and 3,4-DHBA to produce a low amount (0.07 for NBS and 0.08 for NR relative peak area ratio)of norbelladine (**Figure 4A**). To examine if NBS and NR work together for the condensation of tyramine and 3,4-DHBA into norcraugsodine followed by its reduction into norbelladine (**Figure 1**), we tested NBS and NR purified enzymes together in one-step assay and in two-step sequential manner. Our inability to detect the intermediate norcraugsodine through HPLC-MS/MS prompted us to examine the norbelladine production in each assay mixture. We measured the substrates and equal amount of added papaverine (internal standard) in all reaction mixtures for relative quantification of the observed product (**Figures S9A-R**). Norbelladine production was significantly (six-fold, 0.49 relative peak area ratio) higher when both *Np*NBS and *Np*NR were present in a single reaction, compared to assays with *Np*NBS or *Np*NR separately (**Figures 4A, B**; **S9E-J**). Similarly, we observed four-fold higher (0.31 relative peak area ratio) for norbelladine production when *La*NBS and *La*NR were present in a single reaction, compared to assays with *La*NBS or *La*NR individually (**Figures 4A, B**; **S9O-R**). When the reactions were performed in a two-step manner: NBS first followed by NR, we observed two-fold higher norbelladine level (0.92 relative peak area ratio) than when enzymes were together in a single-step for both *Np* and *La* enzymes (**Figures 4A, B**; **S9I, J, Q, R**), which was 12 fold (*Np*) and 8 fold (*La*) higher in comparison to assays with the enzymes individually (**Figures 4A, B** **)**. In the reverse order, stepwise reaction for both species consisted of NR first followed by NBS and yielded lower levels (0.3 relative peak area ratio) of norbelladine than observed with the original sequence (*i.e.*, NBS first followed by NR), but still produced higher amounts of norbelladine compared to single enzyme reactions (**Figures 4A, B**; **S9J, K**). Our results indicate that NBS and NR function together optimally in a sequential manner (NBS first followed by NR) to produce norbelladine. To further confirm the specificity of NR enzyme in norbelladine production, we tested the *Np*TR purified protein individually and together with *Np*NBS in assays containing 3,4-DHBA, tyramine, and NADPH. We observed no norbelladine formation in assays containing *Np*TR enzyme, and assays with both *Np*NBS and *Np*TR in one-step or two-step yielded similar level (0.07 relative peak area ratio) of norbelladine compared to assays containing only *Np*NBS enzyme (**Figures 4A, B**; **S9L-N**). These results confirm the role of NBS and NR together to channel the substrates effectively for the condensation/reduction sequence leading to the formation of norbelladine.

### 3.5 Derivatized norcraugsodine is detected only in assays with both NBS and NR

Direct analysis of norcraugsodine by HPLC-MS/MS or GC-MS was inconclusive (**Figure S11A**). Therefore, we derivatized the norcraugsodine using BSTFA reagent (N,O-bis(trimethylsilyl)trifluoroacetamide) (**Figure S12**). Following derivatization, two signals corresponding to tris-TMS-norcraugsodine (24.79 minutes) and the bis-TMS-3,4-dihydroxybenzaldehyde (9.28 minutes) resulting from the hydrolysis of tris-TMS-norcraugsodine were observed by GC-MS (**Figures S11B-D**; **S12**). *La* and *Np* NBS, and NR purified proteins were tested separately in enzymatic assays containing tyramine, 3,4-DHBA, and NADPH. The resulting assay products were dried, reconstituted in acetonitrile, derivatized using BSTFA, and injected into the GC-MS using electron ionization (EI) at 70 eV. As expected, in assays lacking substrates or enzymes, tris-TMS-norcraugsodine was not detected (**Figures S13A, C**). Similarly, MBP tag alone protein purified from *E. coli* transformed with empty pMAL-c2X vector showed no activity (**Figure S13B**). Surprisingly, we did not detect any tris-TMS-norcraugsodine in assays with single NBS or NR enzyme with or without NADPH (**Figures S13D-K**). However, tris-TMS-norcraugsodine was detected in assays containing both enzymes, in a one-step or two-step fashion, from both *Np* and *La* species (**Figures S13L-O**). In both cases, the detected tris-TMS-norcraugsodine fragmentation pattern matches with the tris-TMS-norcraugsodine standard (**Figure S14**). In the assays with the reverse order of enzymes, *i.e.*, NR first followed by NBS, a very small signal for tris-TMS-norcraugsodine was detected (**Figures S13P, Q**), but the fragmentation pattern did not exactly match with the standard due to proximity with the baseline. As expected, the TR enzyme alone or with NBS did not produce any detectable amounts of tris-TMS-norcraugsodine (**Figures S13R-T**). These results confirm that both NBS and NR are specifically required to harness norcraugsodine and for its efficient reduction into norbelladine.

### 3.6 NBS and NR form a dimer and NBS physically interacts with NR *in planta* and in yeast

**Figure 5:**
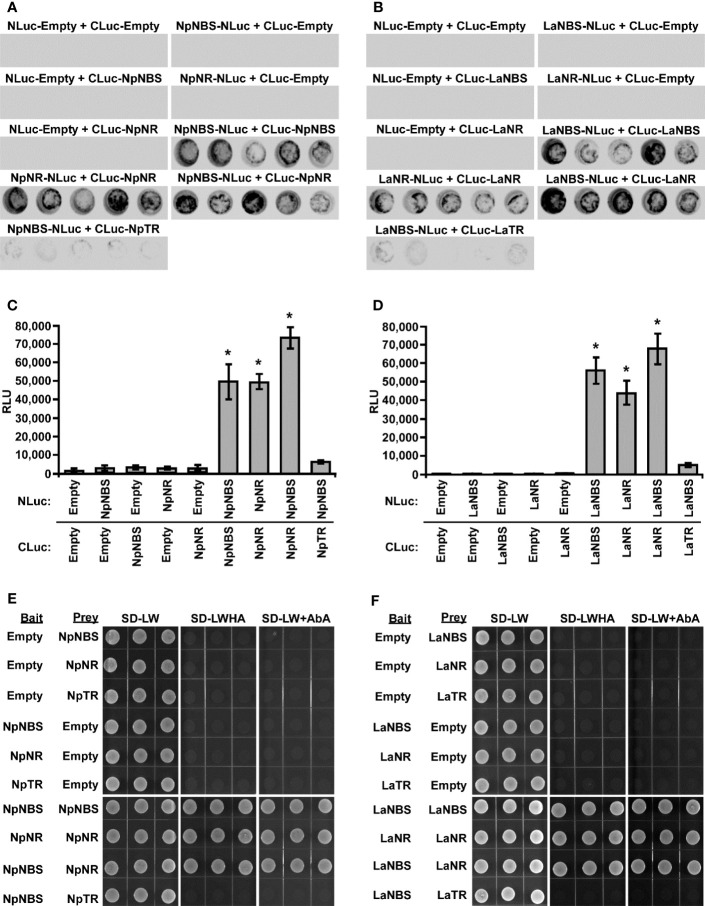
Physical interaction of NBS and NR *in planta* and in Yeast. **(A-D)** The indicated proteins fused to NLuc or CLuc were expressed in leaves of *Nicotiana benthamiana* plants via *Agrobacterium tumefaciens* infection. **(A, B)** The images show LUC images of 96-well microtiter plates containing *N. benthamiana* leaf discs expressing the indicated constructs. **(C, D)** Luciferase activity was quantified as relative luciferase units (RLU) 48 hr post-infiltration. Data are means ± *SE* of three biological repeats. Asterisks indicate a significant difference (Student’s *t* test, *p* < 0.05) relative to empty vector. **(E, F)** Yeast expressing the indicated proteins fused to the GAL4 DNA-binding domain (Bait) or to the GAL4 DNA activation domain (Prey) were grown on synthetically defined (SD) medium lacking Leu and Trp (SD-LW), SD-LW lacking histidine and adenine (SD-LWHA), or SD-LW supplemented with Aureobasidin A (SD-LW+AbA). Empty vectors (EV) were used as negative controls. * = Asterisks indicate a significant difference (Student's t test, p < 0.05).

Previous studies suggest that the norcoclaurine synthase (NCS) proteins assemble as dimers to be catalytically active (Samanani and Facchini, 2002; Samanani et al., 2004; Vimolmangkang et al., 2016). Similarly, the NR protein of *N. pseudonarcissus* was shown to exist as a tetramer through crystal structure of the enzyme (Kilgore et al., 2016b). Based on these observations, we hypothesized that NBS and NR form a dimer which impacts their activity. To explore the ability of using split-luciferase-complementation assay (SLCA) in testing the interactions of NBS and NR in *N. benthamiana* leaves, we first examined the physical interactions between *Np*NBS-*Np*NBS, *La*NBS-*La*NBS, *Np*NR-*Np*NR, and *La*NR-*La*NR. *Np*NBS, *La*NBS, *Np*NR, and *La*NR were fused to the N-terminal half of the luciferase protein (NLuc) and co-expressed via *Agrobacterium* in *N. benthamiana* leaves while *Np*NBS, *La*NBS, *Np*NR, and *La*NR were fused to the C-terminal half of luciferase (CLuc) protein. As negative controls, *Np*NBS-NLuc, *La*NBS-NLuc, *Np*NR-NLuc, and *La*NR-NLuc were co-expressed with CLuc empty vector and CLuc-*Np*NBS, CLuc-*La*NBS, CLuc-*Np*NR, and CLuc-*La*NR were co-expressed with NLuc empty vector (**Figures 5A-D**). Expression of all the fusion proteins was validated by western blot analysis (**Figures S15A, B**). The homodimeric interactions were monitored by measuring luminescence 48 h after agroinfiltration of the tested protein pairs. Co-expression of *Np*NBS-NLuc with CLuc-*Np*NBS, *La*NBS-NLuc with CLuc-*La*NBS, *Np*NR-NLuc with CLuc-*Np*NR, and *La*NR-NLuc with CLuc-*La*NR resulted in emission of significantly higher luminescence compared to the negative controls indicating a physical interaction *in planta* between NBS-NBS and NR-NR fusion proteins (**Figures 5A-D**). One explanation for the production of norbelladine in reactions containing NBS and NR is that NR functions as an enzyme that acts on norcraugsodine produced from tyramine and 3,4-DHBA by NBS. Alternatively, NR may alter the catalytic properties of NBS through allosteric regulation, which allows NBS to form norbelladine, or vice versa, but it remains formally possible that NBS and NR are present together in a metabolon, NR playing a regulating role by guiding the substrates to the imine intermediate followed by reduction to norbelladine. To test the importance of physical interactions for norbelladine biosynthesis, the interactions of *Np*NBS and *La*NBS with full-length *Np*NR and *La*NR were examined in *N. benthamiana* leaves by split-luciferase-complementation-assays. *Np*NBS and *La*NBS were fused to the N-terminal half of the luciferase protein (NLuc) and co-expressed via *Agrobacterium* in *N. benthamiana* leaves along with *Np*NR and *La*NR fused to the C-terminal half of luciferase (CLuc). As a control, *Np*NBS-NLuc and *La*NBS-NLuc were co-expressed with CLuc-*Np*TR and CLuc-*La*TR, respectively. As negative controls, *Np*NBS-NLuc and *La*NBS-NLuc were co-expressed with CLuc empty vector and CLuc-*Np*NR and CLuc-*La*NR were co-expressed with NLuc empty vector (**Figures 5A-D**). Expression of the examined fusion proteins was confirmed by western blot analysis (**Figures S15A, B**). Protein-protein interactions *in planta* were quantified by measurements of luminescence 48 h after agroinfiltration. In agreement with the enzymatic assays, co-expression of *Np*NBS-NLuc with CLuc-*Np*NR and *La*NBS-NLuc with CLuc-*La*NR resulted in emission of significantly higher luminescence compared to the negative controls and the control protein CLuc-*Np*TR and CLuc-*La*TR (**Figures 5A-D**), demonstrating a physical interaction *in planta* between NBS and NR fusion proteins.

To validate the interaction detected *in planta* and to check if the observed interactions are direct, the interactions between NBS-NBS, NR-NR, NBS-NR, and NBS-TR were then examined by yeast two-hybrid system. NBS, NR and TR from both plant species were fused to both bait and prey plasmids. Expression in yeast of bait and prey proteins was confirmed by western blot analysis (**Figures S15C, D**). Similar interactions between NBS-NBS, NR-NR and NBS-NR were observed (**Figures 5E, F**) when the same protein pairs from both plant species were expressed in yeast as bait and prey proteins in agreement with the observed interaction *in planta*. As observed *in planta*, no interaction was found between NBS-TR in yeast (**Figures 5E, F**). Taken together, these results obtained in different experimental systems indicated regulatory interactions between NBS and NR and support the possibility that NBS and NR function as heteromultimeric proteins.

### 3.7 NBS and NR produce norbelladine together *in vivo*

**Figure 6:**
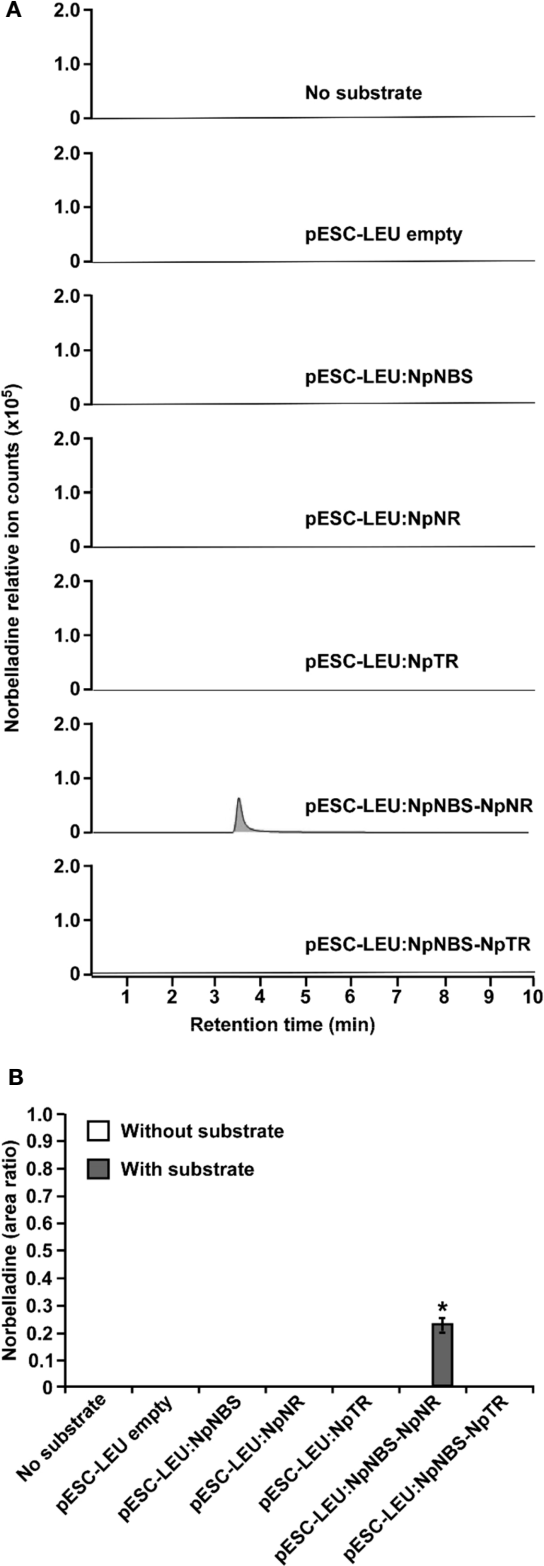
*In vivo* biosynthesis of norbelladine in yeast. HPLC-MS/MS analysis of norbelladine produced in yeast cultures expressing NBS and NR both singly and in combination. **(A)** Extracted ion chromatograms of quantifier MRM transition 260 → 138 m/z showing the product norbelladine in different indicated yeast cultures. **(B)** Comparison and relative quantification of assays shown in **Figure 6A** in triplicate (mean ± SD, n = 3). The norbelladine product profiles in different assays performed were analyzed by HPLC-MS/MS and the obtained amount were relatively quantified using the area ratio of norbelladine produced in the assay to the papaverine internal standard. Data are means ± *SE* of three biological repeats. Asterisks indicate a significant difference (Student’s *t* test, *p* < 0.05) relative to the yeast culture expressing *Np*NBS alone. * = Asterisks indicate a significant difference (Student's t test, p < 0.05).

Functional analysis of NBS and NR via RNAi was not possible because Amaryllidaceae transformation has not been achieved. To demonstrate NBS and NR activity *in vivo*, we reconstituted norbelladine biosynthesis in yeast. Yeast cultures expressing *Np*NBS, *Np*NR, and *Np*TR as single enzymes or *Np*NBS together with *Np*NR or *Np*TR were fed with tyramine and 3,4-DHBA, and norbelladine production was monitored in the yeast cell extracts. We measured the equal amount (1000 mg/L) of added papaverine (internal standard) in all reaction mixtures for relative quantification of the observed norbelladine product. Surprisingly, we did not detect norbelladine in any of the yeast cultures expressing single NBS or NR enzyme (**Figures 6A, B**). However, we detected low levels of norbelladine (0.22 relative peak area ratio) in yeast cultures expressing both NBS and NR (**Figures 6A, B**). This confirms that both enzymes are required for norbelladine production in an *in vivo* model.

### 3.8 NBS and NR colocalize in the cell cytoplasm and nucleus

**Figure 7:**
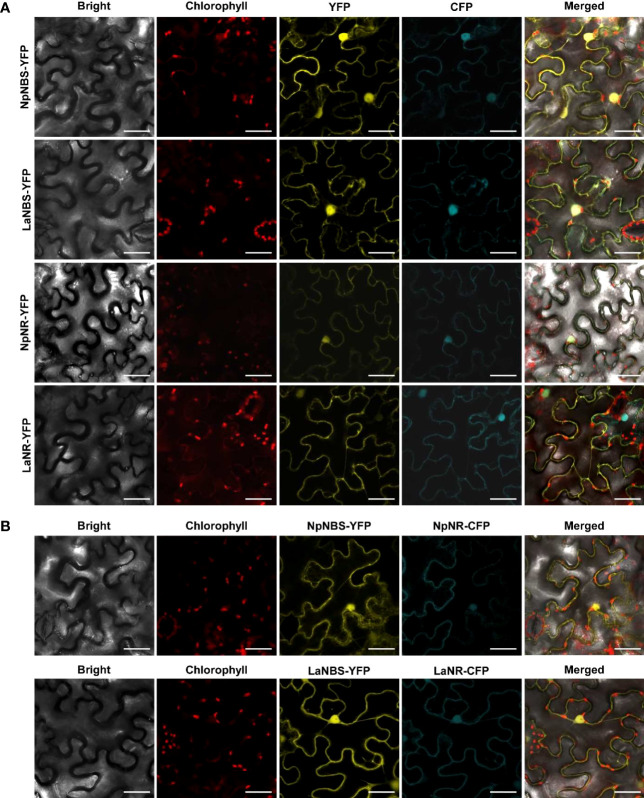
NBS and NR colocalize to the cell cytoplasm and nucleus. **(A)** The indicated fusion proteins were co-expressed with the cyan fluorescent protein (CFP) in *Nicotiana benthamiana* leaves via *Agrobacterium tumefaciens*. After 48 hr, fluorescence was monitored in epidermal cells by confocal microscopy. Bright field, chlorophyll, yellow fluorescent protein (YFP), cyan fluorescent protein (CFP), and merged fluorescence images are shown. Scale bars in images represent 50 μM. **(B)** Confocal micrographs of transiently expressed NBS-YFP and NR-CFP in *Nicotiana benthamiana* leaves showing they colocalize to the cytoplasm and nucleus. Bright field, chlorophyll, yellow fluorescent protein (YFP), cyan fluorescent protein (CFP), and merged fluorescence images are shown. Scale bars in images represent 50 μM.

The NBS protein fused with green fluorescent protein (GFP) from both *L. aestivum* and *N. papyraceus* was recently shown to localize to the cell cytoplasm and nucleus (Tousignant et al., 2022). Similarly, we found NR lacked any predicted signal peptides. To investigate NBS and NR subcellular localization, the *NBS* and *NR* coding regions from both *N. papyraceus* and *L. aestivum* plants were fused upstream to the yellow-fluorescent-protein gene (*YFP*). The *Np*NBS-YFP, *La*NBS-YFP, *Np*NR-YFP, and *La*NR-YFP fusions were transiently expressed in leaves of *N. benthamiana* plants via *A. tumefaciens*, and their localization was monitored by confocal fluorescence microscopy. The cyan fluorescent protein (CFP), which localizes to the cytoplasm and nucleus (Kruse et al., 2010), was used as a control. Expression of all the fusion proteins was validated by western blot (**Figure S16**). As shown in **Figure 7A**, the NBS-YFP and NR-YFP fusion proteins from both plant species localized in the cell cytoplasm and nucleus like CFP. These results suggest that both NBS and NR are distributed to the same nucleocytoplasm cellular compartment. To further confirm the colocalization pattern of NBS and NR, the NR coding regions from both *N. papyraceus* and *L. aestivum* plants were fused upstream to the cyan fluorescent protein (CFP) and co-expressed via *Agrobacterium* in *N. benthamiana* leaves while *Np*NBS and *La*NBS were fused to the YFP, and their localization pattern was monitored by confocal microscopy. Expression of all the fusion proteins was validated by western blot (**Figure S16**). Similar profiles of fluorescence pattern in the cell cytoplasm and nucleus were observed for NBS-YFP and NR-CFP (**Figure 7B**). These results confirm that NBS and NR which lack predicted signal peptides, colocalized to both the cytoplasm and the nucleus of the cell.

### 3.9 *NBS* and *NR* are expressed at high levels in the bulbs of *N. papyraceus* and *L. aestivum*

**Figure 8:**
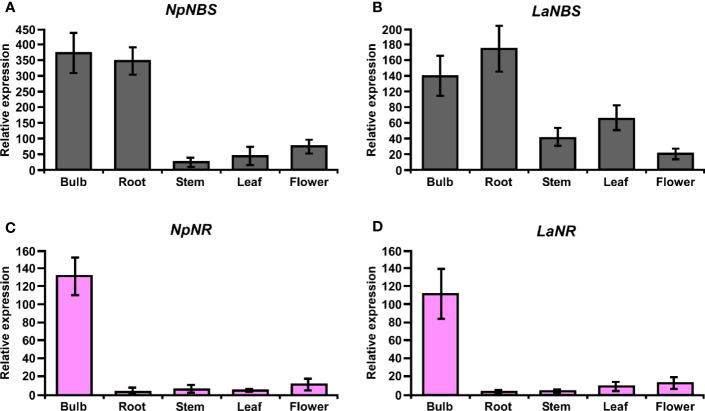
Relative expression of *NBS*, and *NR* in different tissues of *N. papyraceus* and *L. aestivum* using reverse transcription quantitative PCR (RT-qPCR) analysis. Different plant tissues as indicated were harvested after flowering, and *NpNBS* **(A)**, *LaNBS* **(B)**, *NpNR* **(C)**, *LaNR* **(D)** mRNA levels were measured by RT-qPCR analysis relative to expression in leaves. *NpHISTONE* and *LaGAPDH* were used as normalizer. Data are means ± *SE* of three biological repeats.

The expression profiles of the *NBS* and *NR* in different tissues including bulb, root, stem, leaf, and flower were evaluated by using quantitative real-time PCR (qRT-PCR) analysis. The results showed that *NBS* was ubiquitously expressed in all tissues detected, with the highest expression levels in bulb and root (**Figures 8A, B**). The expression patterns of *NpNBS* and *LaNBS* were similar, mRNA accumulated in high amount in bulb and root, and low transcript levels were detected in leaf, stem, and flower (**Figures 8A, B**). The *NR* expression pattern was different. *LaNR* and *NpNR* mRNA specifically accumulated at high levels in bulbs, and in low amount in other tested tissues (**Figures 8C, D**). We further checked the expression pattern of *TR* in different tissues to compare tissue expression profiles between *NR* and *TR* and found that *NpTR* and *LaTR* genes were widely expressed in all the examined tissues (**Figures S17A, B**). These results suggest that the highest transcript levels of *NR* were in bulbs, which parallels with the high expression for *NBS* transcripts in the bulbs. This high expression of *NBS* and *NR* aligns with the higher expression of other AA biosynthetic genes in bulbs of *N. papyraceus* (Hotchandani et al., 2019) and *L. aestivum* (Tousignant et al., 2022).

## 4 Discussion

To date, PR10/Bet v1-like proteins have been directly implicated in the biosynthesis of two alkaloid classes – the AAs and the benzylisoquinoline alkaloids (BIAs) (Dastmalchi, 2021; Morris et al., 2021). Although end-product alkaloids within these two classes are structurally distinct, the pathways have similar biogenic origins. In the BIAs pathway, NCS catalyzes the condensation between dopamine and 4-HPAA to form (*S*)-norcoclaurine. The initiation of AAs’ biosynthesis is proposed to occur via condensation of tyramine and 3,4-DHBA to yield the imine norcraugsodine, which is then reduced to produce norbelladine (**Figure 1**). Our results confirm that the condensation of tyramine and 3,4-DHBA by *La*NBS and *Np*NBS forms norbelladine at low levels, but not norcraugsodine (Singh et al., 2018; Tousignant et al., 2022), despite the absence of a cofactor for the reduction in the reaction mixture. In our enzymatic assays, even the prepared standard solution of norcraugsodine was highly unstable, indicating that norcraugsodine is difficult to detect because of its instability. Still, the imine reduction of norcraugsodine intermediate into norbelladine by NBS is surprising. NBS is part of the PR10/Bet v 1-like enzymes, which are not known to use cofactors for their activity, with few reported exceptions (Jain et al., 2016). Although unlikely, low but enough bacterial components carrying cofactors could remain in the purified protein and help with the reduction. However, in our enzymatic assays, the addition of NADPH to the reaction did not significantly increase the norbelladine production by NBS. Alternatively, *Np*NBS and *La*NBS could possess a reductase activity as found in PR-10 protein *Ca*ARP from chickpea (Jain et al., 2016), but it is improbable as they lack the conserved motifs corresponding to the catalytic signatures of short chain dehydrogenase/reductase (SDR) (Y-X_3_-K) and aldo/keto reductase (AKR) (Y-X_27–30_-K) with the common tyrosine residue (Jain et al., 2016). On the other hand, *NpKA*NR was previously reported to also yield norbelladine following incubation with tyramine, 3,4-DHBA and NADPH, albeit in low yield (Kilgore et al., 2016b). We confirmed that low but detectable amounts of norbelladine can be produced from 3,4-DHBA and tyramine with NR isolated for *N. papyraceus* and *L. aestivum* in enzymatic assays. In theory, other SDR members with imine reduction capability could be able to catalyze this reaction as proposed previously (Roth et al., 2018). In our study, only NR, but not TR, the other identified SDR member, was specifically able to catalyze this reaction. The low yields reported with NBS and NR alone prompted us to hypothesize that they could need to work together to channel the substrates effectively for their condensation into norcraugsodine followed by an immediate reduction into norbelladine. Their interaction could help by improving their catalytic activity, by avoiding degradation of unstable norcraugsodine in decreasing its transit time, and by preventing feedback regulation of norbelladine production.

We used homology modelling and docking studies to model our hypothesis and gain insight of the ligand-enzyme interactions involved in norbelladine synthesis as a two-step reaction. Although performing docking on predicted structures has its limitations, it provides a general scheme on the possible interactions of NBS and NR with their respective ligands that is consistent with the enzymatic reactions they performed. Our proposed model for norbelladine synthesis involves NBS first where the condensation of 3,4-DHBA and tyramine subunits yields an imine/iminium intermediate (Schiff base) (*i.e.*, norcraugsodine), followed with a reduction by NR leading to norbelladine (**Figure 1**). Similarly to the NCS catalyzed reaction (Lichman et al., 2017a; Lichman et al., 2017b), homology modeling and docking results suggest that NBS Tyr68, Lys83 and Glu71 surround the active site and interact with 3,4-DHBA and tyramine, probably playing key roles in norcraugsodine synthesis. In this proposed reaction, Lys83 would transfer a proton from the ammonium group of tyramine to the carbonyl oxygen of 3,4-DHBA, stabilizing a partial positive charge on the carbon atom. Lys83-assisted nucleophilic attack of tyramine amine group to the aldehyde carbonyl would lead to the release of a water molecule from the carbinolamine moiety, generating the imine double bond, in a similar reaction to NCS’s (Lichman et al., 2017b). Finally, norcraugsodine would be formed following an electrophilic attack and deprotonation assisted by the carboxyl moiety of Glu71, acting as a base. Tyr68 could contribute to the electrostatic properties of the active site and by shaping the cavity entrance. Following its synthesis, norcraugsodine would then be reduced to norbelladine inside NR active site with the assistance of NADPH. In SDR enzymes, the catalytic dyad Tyr175 and Lys179 is conserved and have a double role in the active site, the tyrosine serving as a general acid to protonate the substrate keto-group, while the lysine lowers the tyrosine’s pKa to promote proton donation. Noroxomaritidine reduction occurs in a similar structural and chemical arrangement: the substrate ketone is positioned in proximity to Tyr175 and NADPH (Kilgore et al., 2016b). Electrostatic interaction with Lys179 reduces the pKa of Tyr175 to polarize the noroxomaritidine carbonyl group for protonation and hydride transfer, to yield the ketone product with a reduced carbon-carbon double bond (Kilgore et al., 2016b). Similarly, to noroxomaritidine crystal structure in complex with NADPH and tyramine or piperonal, docking results showed that norcraugsodine phenol cycle binds near Phe214, its amine group is positioned close to Tyr173 and NADPH C4, and its dihydroxybenzene binds to Glu224. Lys177 in the proximity of Tyr173 could allow the tyrosine hydroxyl group to serve as a general acid with a hydride transfer from NADPH, leading to norbelladine (*i.e.*, with its reduced imine group). Consistent with reactions catalyzed by the SDR families, docking studies suggest that NADPH, Tyr173 and Lys 177 play key roles in norcraugsodine reduction.

We tested our model pathway of norbelladine biosynthesis using several enzymatic assays. Our results confirmed that when both enzymes are present in the reaction, either in a single-step or in a two-step reaction, significantly higher levels of norbelladine were produced than when using each enzyme separately. The highest yield (12-fold increase) was obtained when the assay was carried out in a two-step manner, with NBS first and NR second. The observation of obtaining more norbelladine in two-step assays than one-step in *in vitro* enzymatic assay is not aligning with our hypothesis that the channelled reactions of the single-step assay would yield highest levels of norbelladine as both the enzymes (NBS and NR) are present together. We propose that the 10 mins boiling step in two-step assays might not have completely inactivated all the NBS protein and the following further 2-hour incubation with NR could have an increased norbelladine production as all the substrates are present in the reactions. Another possibility is that the large MBP tag (42-kDa) present in the fusion protein could be hindering the property of the NBS and NR hetero multimers. Thus, it is possible that NR in inappropriate conformation inhibits NBS or vice versa, but somehow interferes to a lesser extent to that of homo multimers of either or both of NBS and NR. To address this, we attempted to cleave the MBP-tag with Factor Xa protease. Unfortunately, we observed that upon the tag cleavage, both NBS and NR protein precipitated/aggregated, and we were unable to obtain the NBS or NR protein without a MBP tag fusion. Similarly, we found that these two proteins are prone to be in insoluble fraction with a smaller histidine (HIS)-tag. Lower levels of soluble NBS protein with HIS-tag was also reported by Singh et al., 2018. The MBP tag does not dimerize itself as other larger tags such as glutathione S-transferase (GST) (Reuten et al., 2016), and interaction between the two proteins were confirmed in yeast and colocalization in planta using other tag systems. Hence, the possibility of forced dimerization between NBS and NR, and its false implication on the enzymatic assays is unlikely. However, we cannot completely rule out the possibility that the large fusion tag can interfere with proper conformation of the hetero multimers. Future studies that will mutate important amino acids required for the physical interaction of NBS and NR will shed light on how these hetero multimers are formed, and how they regulate biochemical properties of the enzymes. The initial purpose of the two-step assay was to check if we can detect higher norbelladine as we could not detect the intermediate norcraugsodine in our assays. Overall, our results showed that NBS and NR can co-operate to produce norbelladine *in vitro*.

The inability to detect the intermediate norcraugsodine was a major challenge in our study. With very extensive work we found that norcraugsodine is highly unstable and difficult to detect by LC- or GC-MS analysis. However, we could detect the norcraugsodine by GC-MS upon derivatization with BSTFA. Interestingly, our *in vitro* enzymatic assays showed that to detect the derivatized norcraugsodine both enzymes (NBS and NR) required physically present together. We propose a metabolon with NBS and NR proteins, where NR functions as an enzyme that acts immediately on norcraugsodine produced from tyramine and 3,4-DHBA by NBS and converts it to norbelladine. Alternatively, NR may alter the catalytic properties of NBS through allosteric regulation, which allows NBS to form norbelladine, or vice versa. The other possibility is that NBS and NR are present together in a metabolon, and both play a regulatory role together in guiding the substrates to the imine intermediate and keeping the intermediate norcraugsodine stable, followed by immediate reduction to norbelladine. Hence without both proteins norcragusodine is not stabilized and hence cannot be detected. The formation of norcraugsodine in assays s with NBS or NR alone can not be ruled out but if norcraugsodine was indeed produced, it was below detection levels.

*In vivo*, enzymes perform their functions in specific subcellular compartments (Huh et al., 2003). We showed that NBS and NR are colocalized to the cell cytoplasm and nucleus. Their colocalization is consistent with their possible cooperation. We propose that the site of their activity is the cytoplasm as previous studies have shown that *La* tyrosine decarboxylase 1 (TYDC1), which catalyzes the conversion of tyrosine to tyramine, was localized to the cytosol (Wang et al., 2019), as were *O*-methyltransferase 1 from *Lycoris aurea* (*O*MT) and the N4*O*MT from *Lycoris longituba*, which catalyze the *O*-methylation of norbelladine into 4’-*O*-methylnorbelladine (Sun et al., 2018). In addition, there is some evidence of cytoplasmic localization for tyramine and 3,4-DHBA (Nagahashi et al., 1996). Thus, our results support the hypothesis that the early reactions of AAs are biosynthesized in the cytosol. Our gene expression analysis clearly indicates that both NBS and NR from both species are highly expressed in bulb, but only the former in root. This suggests that root has a reduced catalytic efficiency in norbelladine biosynthesis because of the low level of NR expression and unavailability of catalytically active metabolon containing NBS and NR. These results align with the previous work on *Narcissus papyraceus* (Hotchandani et al., 2019) where transcript levels of other AA biosynthetic genes such as TYDC1, TYDC2, PAL2, C4H, C3H and N4*O*MT are very high in the bulb, lower in the roots, and lowest in the leaves. By contrast, at the alkaloid level, the opposite was observed: the bulb tissue has the lowest amount of alkaloids and the leaves and roots the highest. A possible explanation is that the biosynthetic enzymes and/or alkaloids produced in the bulb are being transported to other parts of the plant, leading to fewer alkaloids in the bulb and higher amounts in the roots and leaves (Hotchandani et al., 2019). In Amaryllidaceae plants, we observed combined high expression of both NBS and NR in bulbs of *N. papyraceus* and *L. aestivum*, reinforcing their probable cooperation to produce norbelladine *in vivo* in the bulb tissues, later transported to different parts as the plant grows and develops. Future study with a full set of spatio-temporal profiles of NBS and NR expression together with AA accumulation in *Leucojum aestivum* will shed light on the involvement of the proposed AA biosynthetic genes in the production of the AAs at each developmental stage.

Then, we investigated the possibility that NBS and NR interact together in yeast and *in planta*. First, we observed that both NBS and NR proteins physically interact as homodimers in yeast and *in planta*, suggesting that this conformation is important for their function *in vivo*. Then, our results in yeast suggest that the two enzymes directly interact with each other and that additional plant proteins are not required for this interaction. These results support the hypothesis that NBS and NR work together in a metabolon, where NBS as a homodimer synthesizes the intermediate norcraugsodine, which is readily converted into norbelladine by NR, present as a homodimer in the same protein complex. The formation of metabolons allows the intermediate product to be passed directly from one enzyme to the active site of the subsequent enzyme in the metabolic pathway (Zhang et al., 2017). It is also possible that allosteric regulation of NBS by NR (or vice-versa) could explain the enhanced production of norbelladine when both enzymes interact. NBS or NR could play a chaperone-like role in guiding the folding of the norcraugsodine intermediate or of the second enzyme, as has been previously suggested for PR10/Bet V1-like members (Dastmalchi, 2021; Morris et al., 2021). Metabolons are dynamic and transient protein assemblies that facilitate metabolic reactions within the cells (Dahmani et al., 2023). To date, several metabolons in plants have been suggested to mediate channeling of different substrates, including the TCA cycle (Zhang et al., 2017), the glycolytic pathways, and several pathways of specialized metabolism in plants (Laursen et al., 2016; Camagna et al., 2018; Fujino et al., 2018; Gou et al., 2018). All metabolons involve physical association of different enzymes involved in a specific metabolic pathway and plays a crucial role in cellular metabolism by enhancing reaction rates, protecting unstable intermediates, and minimizing side reactions (Dahmani et al., 2023). In our study we observed all the common features of a metabolon formation like enzyme-enzyme complex, detection of substrate channels, presence of the enzymes in same cellular compartments. Our results clearly showed that NBS and NR are present as homo- and heteromeric complexes, which protect and channel the unstable intermediate norcraugsodine. Furthermore, their interactions are direct, and localization to same nucleocytoplasmic compartments. We propose that, as AAs biosynthesis in Amaryllidaceae plant cells is a very complex process due to the number of subcellular organelles in plant cell, and the number of isoforms of specific enzymes they contain, and the sheer range of metabolites they form, possible formation of different metabolons will be ideal to regulate mechanisms to rapidly and frequently alter metabolic fluxes in response to specific demands or challenges. Future studies involving X-ray crystallography, electron microscopy, and development of artificial intelligence (AI) for accurate prediction of complex structures, will help us to better understand how different sequential enzymes in AAs biosynthesis form structural-functional complexes and how this metabolon is regulated.

Finally, we used a yeast system to express NBS alone, NR alone, or both NBS/NR to show that their interaction is essential to generate higher levels of norbelladine. Our results confirmed that when both NBS and NR are present together, the yeast produces detectable levels of norbelladine, as compared to the absence of detectable norbelladine when each protein was present alone or when the yeast was simply fed with tyramine and 3,4-dihydroxybenzaldehyde. Future studies with metabolic engineering of yeast with the complete elucidated pathway for galanthamine biosynthesis will help us better understand how this pathway is regulated and how to increase the production of norbelladine in heterologous systems.

In conclusion, our study establishes that NBS and NR cooperatively catalyze the biosynthesis of norcraugsodine and its reduction into norbelladine. We show that the two enzymes localize to both the cytoplasm and nucleus, are expressed at high levels in bulbs, and physically interact with each other in yeast and *in planta*. This study unravels the reactions involved in the first key steps of the biosynthesis of all AAs. Deciphering norbelladine synthesis will facilitate the development of the biosynthetic tools required to produce AAs *in vitro* and help fight human diseases, for example via the biosynthesis of galanthamine in heterologous hosts to treat the symptoms of Alzheimer’s disease.

## Data availability statement

The datasets presented in this study can be found in online repositories. The names of the repository/repositories and accession number(s) can be found in the article/**Supplementary Material**.

## Author contributions

IDP conceived, designed, and supervised the study. BBM designed and performed most of the experiments. SEG performed all the HPLC-MS/MS analysis. NM carried out the protein modelling and docking experiments. SR carried out the derivatization of norcraugsodine and GC-MS analysis. BBM, NM, and IDP wrote the manuscript with input from others. All authors read and approved the final version of the manuscript. All authors contributed to the article.
